# Attenuated Impact of Gestational Diabetes Mellitus (GDM) With Hypertensive Disorders of Pregnancy (HDP) on Adverse Pregnancy Outcomes in Twin Versus Singleton Gestations: A Single‐Center Real‐World Study

**DOI:** 10.1155/jdr/3096949

**Published:** 2026-08-16

**Authors:** Jing Bao, Ping Guan

**Affiliations:** ^1^ Department of Obstetrics, Maternal and Child Health Hospital of Hubei Province, Wuhan City, Hubei Province, China

**Keywords:** GDM, HDP, perinatal outcomes, pregnancy, twin

## Abstract

**Objective:**

Gestational diabetes mellitus (GDM) and hypertensive disorders of pregnancy (HDP) are two of the most prevalent pregnancy complications affecting both singleton and twin pregnancies worldwide. This study specifically investigates the combined effect of concurrent GDM and HDP on maternal and perinatal outcomes among twin gestation populations.

**Methods:**

This retrospective single‐center study enrolled 132,314 pregnant people who delivered at ≥ 20 weeks of gestation as the eligible study population, including 918 stillbirth cases, 2803 live twin births, and 128,593 live singleton births. All included cases met core inclusion and exclusion criteria. The total population was categorized into four subgroups according to the presence of HDP and/or GDM: non–HDP‐GDM, HDP only, GDM only, and concurrent HDP‐GDM. The primary analysis focused on comparing perinatal outcomes between twin and singleton pregnancies within each of the four subgroups to evaluate the effect of HDP, GDM, and their co‐occurrence on perinatal outcomes across different pregnancy types.

**Results:**

Among the 132,314 pregnant people included in this study, the stillbirth rate in twin pregnancies was higher than that in singleton pregnancies (1.79% vs. 0.67%, *p* < 0.001). Distinct differences in maternal and neonatal complication profiles were observed between singleton and twin pregnancies, and this discrepancy persisted across both term and preterm subgroups. The incidence of HDP and GDM in twin pregnancies was 23.0% and 21.0%, respectively. Across all subgroups, twin pregnancies were associated with preterm birth (PTB), cesarean section (CS), and anemia. Interestingly, the number of complications showing comparable occurrence patterns between singleton and twin pregnancies was 3 in the non–HDP‐GDM group, 6 in the HDP group, 8 in the GDM group, and 15 in the HDP‐GDM group. Notably, the positive association between HDP/GDM and adverse outcomes observed in singleton pregnancies was not equivalently present in twin pregnancies. In singleton pregnancies, the combined effect of HDP and GDM on adverse outcomes could either synergistically amplify or counteract (attenuate) their individual impacts, depending on whether their individual effects were congruent—a pattern with synergistic amplification when individual risks were congruent. In contrast, the combined influence of HDP and GDM showed limited association with adverse outcomes in twin pregnancies.

**Conclusions:**

Although the individual incidence of HDP and GDM is higher in twin pregnancies, their combined effect on adverse perinatal outcomes is attenuated in twin populations. This retrospective single‐center finding offers reference for individualized risk assessment of twin pregnancies.

## 1. Introduction

Diabetes is a disease that severely endangers public health, among which gestational diabetes mellitus (GDM) is a condition affecting specific populations during a special phase, which is the most prevalent metabolic complication of pregnancy [1]. With the rising incidence of twin pregnancies and the unique physiological environment of such pregnancies, the clinical and complication profiles of GDM demand heightened focus [2]. Twin gestations are associated with substantially elevated risks of obstetrical complications for both mother and fetus, including GDM, hypertensive disorders of pregnancy (HDP) and severe neonatal morbidity [3, 4]. Therefore, the management of GDM in twin pregnancies needs to take into account the effects of coexisting HDP. Although preliminary evidence indicates distinct etiologies of adverse outcomes between twin and singleton gestations, current clinical guidelines for twin pregnancy management remain largely derived from singleton‐based evidence [5]. Current systematic reviews and meta‐analyses indicate divergent and inconclusive evidence regarding the impact of GDM/HDP on maternal‐fetal outcomes in both twin and singleton pregnancies [3, 6]. To mitigate severe complications, targeted evidence must be generated to implement twin‐specific screening protocols and clinical management strategies [7]. Therefore, analyzing the differences in major gestational comorbidities between singleton and twin pregnancies based on real‐world data from large clinical samples is of significant clinical importance.

It is evident that singleton pregnancies complicated by GDM or HDP consistently demonstrate adverse outcomes [8]. Twin gestation has been identified as an independent risk factor for the development of both HDP and GDM, primarily attributable to increased placental volume and metabolic demand [6, 9]. The placenta has been demonstrated to play a pivotal role in the pathophysiological mechanisms that underpin gestational diabetes, hypertension, and fetal growth abnormalities [10]. The risk of adverse neonatal outcomes in twin pregnancies with GDM may be elevated or comparable to control groups [11–13]. Conversely, emerging evidence paradoxically suggests a potential protective effect of GDM in twins against specific perinatal complications, including neonatal asphyxia and perinatal mortality [14]. These conflicting findings may be constrained by limited sample sizes and failure to stratify for comorbidities—particularly concurrent GDM and HDP—thereby obscuring the distinct impact of plurality on GDM‐associated outcomes [13, 15].

Clinically noteworthy, GDM, HDP, and twin pregnancy share common risk factors including elevated body mass index and advanced maternal age, leading to a relatively higher prevalence of concurrent GDM and HDP among twin pregnancies [16]. Accordingly, exploring its clinical characteristics and optimizing diagnostic and therapeutic strategies carries important clinical implications. Some scholars hold that the combined adverse effects of the two disorders simply superimpose in twin gestations. Based on existing literature and clinical theories, we hypothesized that concomitant GDM and HDP exert synergistically aggravated adverse effects on singleton pregnancies, whereas such combined impacts may be attenuated in twin pregnancies due to their distinctive physiological features.

Against this background, this real‐world study is aimed at comparing the incidences of GDM and HDP in twin pregnancies with that in singleton pregnancies. Perinatal outcome risks were further assessed across four subgroups stratified by GDM and HDP comorbidity status. Through this study, it is expected to clarify the distinct impacts of GDM and HDP on singleton and twin pregnancies, provide more sufficient evidence for the management of twin pregnancies, and guide the precise management of pregnant women with both GDM and HDP.

## 2. Methods

### 2.1. Study Population

In this study, we conducted a retrospective analysis of medical records from the Obstetrics Department of Hubei Provincial Maternity and Child Health Hospital (Wuhan, China). This study was conducted from June 2023 to June 2024. A total of 132,314 eligible pregnant women with deliveries were enrolled, comprising 918 cases with stillbirths, 2803 with live twin births, and 128,593 with live singleton births. The inclusion criteria are included as follows: (1) Received prenatal care at Hubei Provincial Maternal and Child Health Hospital and delivered in the obstetrics department; (2) delivery period from January 1, 2017 to June 30, 2022; (3) gestational age ≥ 20 weeks at delivery; (4) the pregnant women included in this study consist of those with natural conception and artificial insemination; (5) with essential data needed in the present study. The study included all gravidas who gave birth at ≥ 20 gestational weeks in our department between January 1, 2017, and June 30, 2022. The exclusion criteria included: stillbirth and termination of pregnancy for fetal anomalies Aa a result, a total of 132,314 eligible pregnant women with deliveries were enrolled, comprising 918 cases with stillbirths, 2803 with live twin births, and 128,593 with live singleton births.

**Figure 1:**
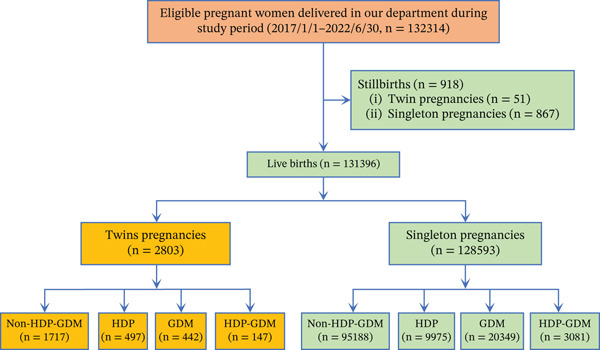
The flowchart of this study. Selection of the study cohort from deliveries at the Obstetrics Department of Hubei Provincial Maternity and Child Health Hospital (Wuhan, China) between January 1, 2017 and June 30, 2022, culminating in the analysis of 128,593 singleton and 2803 twin live births, categorized into four HDP/GDM exposure groups.

Furthermore, according to whether pregnant women have GDM or HDP, the study population was divided into non–HDP‐GDM group, HDP group, GDM group, and HDP‐GDM group. The non–HDP‐GDM group was defined as pregnant women without HDP or GDM. The HDP group was defined as pregnancies complicated with HDP but without GDM. The GDM group was defined as pregnancies with GDM but without HDP. The HDP‐GDM group was defined as pregnancies with both HDP and GDM. Among twin pregnancies, the number of participants in the non–HDP‐GDM, HDP, GDM and HDP‐GDM groups was 1717, 47, 442, and 147, respectively. For singleton pregnancies, the corresponding counts were 95,188; 995; 20,349; and 3081. The research flowchart was presented in Figure 1.

The observational research protocol for this study was approved by the Medical Ethics Committee of Maternal and Child Health Hospital of Hubei Province. The study was conducted in accordance with the local legislation and institutional requirements. The ethics committee/institutional review board waived the requirement of written informed consent for participation from the participants or the participants′ legal guardians because it was a retrospectively real‐world study. We only extracted relevant clinical information, did not affect the treatment and health of patients, and the patients′ privacy was protected.

### 2.2. Outcome Measures

Maternal characteristics, such as maternal age, ethnicity, history of cesarean delivery, number of fetuses, gestational week, and delivery mode, were retrieved from the hospital′s information system. GDM was diagnosed according to the 2010 criteria issued by the International Association of Diabetes and Pregnancy Study Groups (IADPSG) [17]. A 75‐g oral glucose tolerance test was performed at 24–28 weeks of gestation. GDM was confirmed if any blood glucose value reached 5.1 mmol/L at fasting, 10.0 mmol/L at 1 h or 8.5 mmol/L at 2 h after glucose loading. HDP were classified and diagnosed based on the 2021 guidelines of the International Society for the Study of Hypertension in Pregnancy (ISSHP) [18]. Proteinuria is no longer an essential diagnostic indicator. Disease subtypes were determined by blood pressure levels and organ damages involving liver, kidney, hematological system, and fetus.

Diagnoses were confirmed using the 10th Revision of the International Classification of Diseases (ICD‐10) coding system from electronic medical records, encompassing fetal weight‐related conditions (macrosomia and low birth weight [LBW]) and pregnancy complications such as HDP, GDM, PTB, placenta previa, placental abruption, and cord prolapse. HDP comprised chronic hypertension, gestational hypertension (GH), preeclampsia (PE), and eclampsia. Further information on short‐term perinatal outcomes, such as intrauterine distress, neonatal asphyxia, uterine rupture, puerperal infection, and postpartum hemorrhage (PPH) were also collected.

### 2.3. Statistical Analysis

All analyses were conducted using SPSS Version 23.0 (IBM, Armonk, New York, United States). Quantitative variables were expressed as mean values ± SD. Qualitative variables were expressed as absolute and relative frequencies. For comparisons of continuous variables between the two groups, an independent‐samples *t*‐test was used when the data met the assumptions of normality and homogeneity of variance; otherwise, the nonparametric Mann–Whitney *U* test was applied. The chi‐square test was employed for categorical variables. Odds ratios (ORs) were calculated to evaluate the association between exposure factors and disease risk. Of note, all ORs reported herein were crude values and have not been adjusted for major confounding factors, including maternal age, parity, assisted reproductive technology, and body mass index. Statistical significance was considered when *p* < 0.05.

## 3. Results

### 3.1. Incidence Differences in Pregnancy Complications and Perinatal Outcomes Between Singleton and Twin Pregnancies

Among the 132,314 pregnant women included in the study, the stillbirth rate in twin pregnancies was found to be 1.79% versus singleton pregnancies, which was found to be 0.67%. The proportion of live twin pregnancies with assisted reproduction technology (ART) was 24.7% versus singleton pregnancies, which was 2.5%. Differences between twin pregnancies and singleton pregnancies were identified in 23 variables. Among them, 16 variables (such as GDM and HDP) were demonstrated in twin pregnancies, and the remaining seven variables are shown in Table 1. Perinatal outcome risks in term twin pregnancies were comparable to those in overall pregnancies. In premature twin pregnancies, certain risks remained stable (Tables S1, S2, and S3).

**Table 1:** Basic information of the study population and overall comparison of pregnant complications and outcomes between singleton and twin pregnancies.

Variables	Total pregnancies (*n*, %)	Groups			
		Singleton pregnancies (*n*, %)	Twin pregnancies (*n*, %)	*p*	OR (95% CI) (twin vs. singleton)^a^
**Still births**	*n* = 132,314	*n* = 129,460	*n* = 2854		
Yes	918 (0.69)	867 (0.67)	51 (1.79)	< 0.001^A^	2.699 (2.030–3.588)^A^
No	131,396 (99.31)	128,593 (97.87)	2803 (2.13)		
**Live births**	*n* = 131,396	*n* = 128,593	*n* = 2803		
Gestational age (weeks)	38.35	35.11	38.41	< 0.001	
ART	3947 (3.0)	3253 (2.5)	694 (24.8)	< 0.001^A^	12.549 (11.444–13.760)^A^
HDP	13,700 (10.4)	13,056 (10.2)	644 (23.0)	< 0.001^A^	2.640 (2.413–2.888)^A^
GDM	24,019 (18.3)	23,430 (18.2)	589 (21.0)	< 0.001^A^	1.194 (1.089–1.309)^A^
Placenta previa	2984 (2.3)	2887 (2.2)	96 (3.4)	< 0.001^A^	1.544 (1.256–1.899)^A^
PROM	23,778 (18.1)	23,427 (18.2)	351 (12.5)	< 0.001^B^	0.643 (0.564–0.719)^B^
Placental abruption	1059 (0.8)	1010 (0.8)	49 (1.7)	< 0.001^A^	2.248 (1.683–3.001)^A^
Placental implantation	966 (0.7)	919 (0.7)	47 (1.7)	< 0.001^A^	2.369 (1.763–3.184)^A^
Cord protrusion	192 (0.1)	186 (0.1)	6 (0.2)	0.341	1.481 (0.656–3.342)
Cord entanglement	14,977 (11.4)	14,825 (11.5)	152 (5.4)	< 0.001^B^	0.440 (0.373–0.519)^B^
Cord torsion	9691 (7.4)	9579 (7.4)	112 (4.0)	< 0.001^B^	0.517 (0.428–0.625)^B^
Rupture of uterus	1555 (1.2)	1523 (1.2)	32 (1.1)	0.836	0.964 (0.678–1.370)
Postpartum embolism	69 (0.1)	61 (0.)	8 (0.3)	< 0.001^A^	6.031 (2.883–12.615)^A^
Postpartum thrombosis	365 (0.3)	347 (0.3)	18 (0.6)	< 0.001^A^	2.389 (1.485–3.842)^A^
PTB	10,799 (8.2)	8957 (7.0)	1842 (65.7)	< 0.001^A^	25.602 (23.612–27.759)^A^
Macrosomia	5446 (4.1)	5444 (4.2)	2 (0.1)	< 0.001^B^	0.016 (0.004–0.065)^B^
LBW	384 (0.3)	357 (0.3)	27 (1.0)	< 0.001^A^	3.494 (2.358–5.176)^A^
Neonatal asphyxia	755 (0.6)	681 (0.5)	74 (2.6)	< 0.001^A^	5.093 (3.995–6.493)^A^
PPH	4924 (3.7)	4790 (3.7)	134 (4.8)	< 0.001^A^	1.298 (1.088–1.547)^A^
CS	63,917 (48.6)	61,213 (47.6)	2704 (96.5)	< 0.001^A^	30.065 (24.594–36.753)^A^
Intrauterine distress	8811 (6.7)	8766 (6.8)	45 (1.6)	< 0.001^B^	0.223 (0.166–0.300)^B^
FGR	196 (0.1)	168 (0.1)	28 (1.0)	< 0.001^A^	7.713 (5.161–11.528)^A^
SGA	249 (0.2)	225 (0.2)	24 (0.9)	< 0.001^A^	4.927 (3.229–7.518)^A^
Anemia	12,345 (9.4)	11,864 (9.2)	481 (17.2)	< 0.001^A^	2.038 (1.844–2.252)^A^
Polyhydramnios	2027 (1.5)	1949 (1.5)	78 (2.7)	< 0.001^A^	1.847 (1.464–2.331)^A^
Oligohydramnios	4487 (3.4)	4456 (3.4)	31 (1.1)	< 0.001^B^	0.314 (0.220–0.448)^B^
Amniotic fluid contamination	888 (0.7)	882 (0.7)	6 (0.2)	< 0.001^B^	0.311 (0.139–0.694)^B^

*Note:* Odds ratios (ORs) and 95% confidence intervals (CIs) were derived from unadjusted analyses.

Abbreviations: ART, assisted reproductive technology; CS, cesarean section; FGR, fetal growth restriction; GDM, gestational diabetes mellitus; HDP, hypertensive disorders of pregnancy; LBW, low birth weight; OR, odds ratio (unadjusted, crude OR); PPH, postpartum hemorrhage; PROM, premature rupture of membranes; PTB, preterm births; SGA, small for gestational age.

^a^Uppercase letter “A” indicates significantly increased risk in twin versus singleton pregnancies (*p* < 0.05); “B” indicates significantly decreased risk.

**Figure 2:**
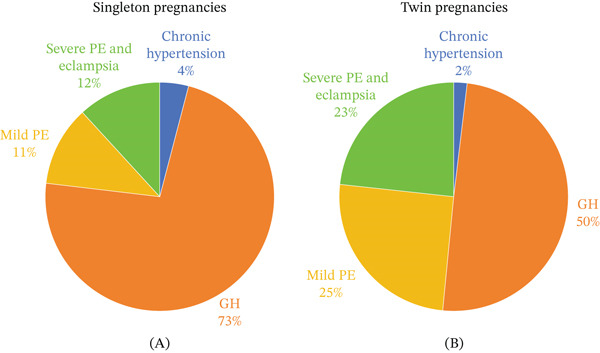
Differences in the distribution of HDP subgroups in singleton and twin pregnancies. (A) The gestational hypertension (GH) is the main type in singleton pregnancies with HDP. (B) The proportion of preeclampsia (PE) significantly increases in twin pregnancies with HDP.

The unadjusted incidence of HDP in twin pregnancies was 260% higher than that in singletons (unadjusted, crude OR, Table 1). Subgroup analysis indicated that the incidence of chronic hypertension was comparable between twin and singleton pregnancies. The proportion of PE and eclampsia in twin pregnancies was higher than that in singletons. The risk of mild PE, severe PE, and eclampsia in twin pregnancies was 25% and 23%, respectively, whereas it was 11% and 12% in singletons, respectively (Figure 2, Table S4). The risk of GDM was 21.0% and 18.2% in twin pregnancies and singletons, respectively (*p* < 0.001) (Table 1). In addition, subgroup analysis showed that the incidence of ketosis was 0.2% and 0.1% in twin and singleton GDM pregnancies, respectively (*p* = 0.355) (Table S5).

### 3.2. Effect Differences of HDP/GDM on Singleton Versus Twin Pregnancy Outcomes

Substantial differences were observed in the distribution of HDP, GDM, concurrent HDP, and GDM, and non–HDP‐GDM status between twin and singleton pregnancy populations (*p* < 0.001). Specifically, the proportions of HDP and concurrent HDP‐GDM among twin pregnancies were 17.7% and 5.2%, respectively, whereas the corresponding proportions among singleton pregnancies were 7.8% and 2.4% (Table 2).

**Table 2:** The differences in the proportion of four groups between singleton and twin pregnancies with HDP/GDM.

HDP/GDM groups	Total pregnancies (*n* = 131, 396; %)	Groups		
		Singleton pregnancies (*n* = 128, 593; %)	Twin pregnancies (*n* = 2803, %)	*p*
Non–HDP‐GDM group	96905 (73.8)	95188 (74.0)	1717 (61.3)	< 0.001
HDP group	10472 (8.0)	9975 (7.8)	497 (17.7)	
GDM group	20,791 (15.8)	20,349 (15.8)	442 (15.8)	
HDP‐GDM group	3228 (2.4)	3081 (2.4)	147 (5.2)	

Abbreviations: GDM, gestational diabetes mellitus; HDP, hypertensive disorders of pregnancy.

In the non–HDP‐GDM group, none of the pregnancies were affected by HDP or GDM. When evaluating complication profiles across pregnancy types, twin pregnancies were associated with 13 complications including placenta previa, placental abruption, placental implantation, postpartum embolism, postpartum thrombosis, PTB, LBW, neonatal asphyxia, CS, fetal growth restriction (FGR), small for gestational age (SGA), anemia, and polyhydramnios. Seven other complications showed higher frequency in singleton pregnancies: premature rupture of membranes (PROM), cord entanglement, cord torsion, macrosomia, intrauterine distress, oligohydramnios, and amniotic fluid contamination. (Table 3, Table S6).

**Table 3:** The difference in risk between twins and singletons under different HDP and GDM effects (with risk in singleton as the reference).

Variables	Groups			
	Non–HDP‐GDM group OR (95% CI)	HDP group OR (95% CI)	GDM group OR (95% CI)	HDP‐GDM group OR (95% CI)
Placenta previa	1.447 (1.105‐1–895)^A^	2.239 (1.414–3.716)^A^	1.403 (0.832–2.366)	2.431 (1.028–5.751)^A^
PROM	0.661 (0.575–0.760)^B^	0.613 (0.459–0.820)^B^	0.721 (0.540–0.963)^B^	0.625 (0.357–1.094)
Placental abruption	2.409 (1.656–3.505)^A^	1.173 (0.545–2.527)	2.162 (1.058–4.421)^A^	2.430 (0.949–6.223)
Placental implantation	2.420 (1.629–3.594)^A^	2.748 (1.494–5.056)^A^	1.646 (0.770–3.519)	1.314 (0.312–5.537)
Cord protrusion	1.805 (0.666–4.891)	UA	2.251 (0.543–9.337)	UA
Cord entanglement	0.415 (0.334–0.517)^B^	0.603 (0.419–0.868)^B^	0.373 (0.248–0.559)^B^	0.530 (0.276–1.016)
Cord torsion	0.517 (0.404–0.661)^B^	0.573 (0.368–0.893)^B^	0.548 (0.353–0.852)^B^	0.250 (0.079–0.790)^B^
Rupture of uterus	0.771 (0.469–1.265)	1.768 (0.706–4.431)	1.289 (0.634–2.618)	2.050 (0.619–6.784)
Postpartum embolism	6.005 (2.138–16.866)^A^	3.350 (0.403–27.878)	3.294 (0.432–25.102)	10.610 (1.928–58.403)+
Postpartum thrombosis	2.495 (1.322–4.709)^A^	0.802 (0.109–5.934)	2.696 (1.089–6.675)^A^	3.255 (0.728–14.559)
PTB	27.182 (24.552–30.093)^A^	16.182 (13.250–19.764)^A^	26.556 (21.568–32.697)^A^	13.746 (9.505–19.878)^A^
Macrosomia	0.026 (0.007–0.105)^B^	UA	UA	UA
LBW	5.368 (3.345–8.616)^A^	1.132 (0.412–3.111)	2.273 (0.710–7.271)	0.775 (0.105–5.741)
Neonatal asphyxia	6.290 (4.691–8.434)^A^	2.418 (1.247–4.687)^A^	5.117 (2.726–9.603)^A^	1.200 (0.286–5.039)
PPH	1.228 (0.973–1.550)	1.373 (0.938–2.069)	1.277 (0.819–1.991)	1.234 (0.565–2.693)
CS	27.231 (21.565–34.385)^A^	30.936 (16.996–56.308)^A^	36.407 (19.999–66.277)^A^	24.317 (7.732–76.472)^A^
Intrauterine distress	0.159 (0.102–0.248)^B^	0.228 (0.117–0.442)^B^	0.421 (0.237–0.750)^B^	0.407 (0.149–1.112)
FGR	12.897 (7.342–22.658)^A^	2.797 (1.328–5.891)^A^	5.556 (1.671–18.469)^A^	2.111 (0.489–9.118)
SGA	6.139 (3.376–11.165)^A^	3.084 (1.385–6.865)^A^	4.313 (1.541–12.067)^A^	0.775 (0.105–5.741)
Anemia	1.935 (1.704–2.197)^A^	2.682 (2.126–3.382)^A^	2.062 (1.577–2.697)^A^	2.456 (1.528–3.949)^A^
Polyhydramnios	1.792 (1.308–2.455)^A^	2.195 (1.255–3.838)^A^	1.908 (1.179–3.086)^A^	1.125 (0.348–3.639)
Oligohydramnios	0.284 (0.179–0.453)^B^	0.211 (0.079–0.568)^B^	0.676 (0.334–1.368)	0.220 (0.030–1.590)
Amniotic fluid contamination	0.335 (0.125–0.898)^B^	UA	UA	2.223 (0.513–9.634)

*Note:* Uppercase letter “A” indicates significantly increased risk in twin versus singleton pregnancies (*p* < 0.05); “B” indicates significantly decreased risk. Odds ratios (ORs) and 95% confidence intervals (CIs) were derived from unadjusted analyses.

Abbreviations: CS, cesarean section; FGR, fetal growth restriction; LBW, low birth weight; OR, odds ratio (unadjusted, crude OR); PPH, postpartum hemorrhage; PROM, premature rupture of membranes; PTB, preterm births; SGA, small for gestational age; UA, due to the case being 0, the OR value was unavailable.

In the HDP group, all pregnancies were affected by HDP but not by GDM. Compared with singleton pregnancies. Within the population, nine complications were linked to twin pregnancies: placenta previa, placental implantation, PTB, neonatal asphyxia, CS, FGR, SGA, anemia, and polyhydramnios. Five other complications occurred more frequently in singleton pregnancies: PROM, cord entanglement, cord torsion, intrauterine distress, and oligohydramnios. For the remaining six complications (placental abruption, uterine rupture, postpartum embolism, postpartum thrombosis, LBW, and PPH), between‐group incidence patterns were comparable (Table 3, Table S7).

In the GDM group, all pregnancies were affected by GDM but not by HDP. Within this population, nine complications were linked to twin pregnancies: placental abruption, postpartum thrombosis, PTB, neonatal asphyxia, CS, FGR, SGA, anemia, and polyhydramnios. Four other complications occurred more frequently in singleton pregnancies. For the remaining eight complications (placenta previa, placental implantation, cord protrusion, uterine rupture, postpartum embolism, LBW, PPH, oligohydramnios, and amniotic fluid contamination), between‐group incidence patterns were comparable (Table 3, Table S8).

In the HDP‐GDM group, all pregnancies were affected by both HDP and GDM. Compared with singleton pregnancies, twin pregnancies exhibited elevated risk of five complications (placenta previa, postpartum embolism, PTB, CS, and anemia), while showing reduced risk of one complication (cord torsion). No significant difference was observed in the risk of the remaining 15 complications (Table 3, Table S9).

In the HDP‐GDM group, all pregnancies were affected by both HDP and GDM. Within this population, five complications were linked to twin pregnancies: placenta previa, postpartum embolism, PTB, CS, and anemia. One other complication occurred more frequently in singleton pregnancies: cord torsion. For the remaining 15 complications, between‐group incidence patterns were comparable (Table 3, Table S9).

Across all subgroup populations, three comorbidities were linked to twin pregnancies: PTB, CS, and anemia. One other comorbidity occurred more frequently in singleton pregnancies: cord torsion. For two comorbidities (uterine rupture and PPH), incidence patterns were comparable between the two groups. The number of complications with comparable incidence patterns between singleton and twin pregnancies was 3 in the non–HDP‐GDM group, 6 in the HDP group, 8 in the GDM group, and 15 in the HDP‐GDM group (Table 3).

### 3.3. The Differences in Adverse Outcomes Among Different HDP/GDM Subgroups in the Singleton Pregnancies

Among singleton pregnancies (*n* = 128,593), differences in perinatal complication risks were observed across the four groups, except for cord protrusion and amniotic fluid contamination (Table S10). Compared with the non–HDP‐GDM group, the HDP group showed positive association with the risk of 10 complications (placental abruption, placental implantation, PTB, LBW, neonatal asphyxia, PPH, CS, intrauterine stress, FGR, and SGA) and reduced risk of five complications (placenta previa, PROM, cord angle, uterine rupture, and anemia). Relative to the non–HDP‐GDM group, the GDM group exhibited risk of 12 complications (placental abruption, placental implantation, cord angle, cord torsion, uterine rupture, postpartum thrombosis, PTB, LBW, CS, FGR, SGA, and polyhydramnios). Compared with the non–HDP‐GDM group, the HDP‐GDM group demonstrated risk of 11 complications (placental abruption, placental implantation, postpartum embolism, postpartum thrombosis, PTB, LBW, neonatal asphyxia, CS, FGR, SGA, and polyhydramnios).

Notably, when comparing to the non–HDP‐GDM group, a synergistic effect was observed in the HDP‐GDM combined group for singleton pregnancies: when the perinatal complication risks in the HDP and GDM groups were congruent (i.e., both increased for placental abruption, placental implantation, PTB, LBW, CS, FGR, SGA; or both decreased for PROM, and anemia), the magnitude of risk increase or decrease in the HDP‐GDM group was more pronounced. Conversely, when the perinatal complication risks in the HDP and GDM groups diverged or differed, the synergistic effect depended on the relative strength of each risk factor (Table 4).

**Table 4:** The difference in singleton outcomes between those affected solely by HDP or GDM, and those affected simultaneously by HDP and GDM (with risk in non–HDP‐GDM singleton pregnancies as the reference).

Variables	Groups			
	Non–HDP‐GDM group	HPP group OR (95% CI)	GDM group OR (95% CI)	HDP‐GDM group OR (95% CI)
Placenta previa	Ref	0.744 (0.636–0.871)^B^	1.075 (0.974–1.186)	0.751 (0.571–0.989)^B^
PROM^B^	Ref^B^	0.827 (0.783–0.875)^B^	0.803 (0.771–0.837)^B^	0.716 (0.647–0.793)^B^
Placental abruption^A^	Ref^A^	1.708 (1.405–2.076)^A^	1.195 (1.010–1.414)^A^	2.032 (1.494–2.762)^A^
Placental implantation	Ref	1.417 (1.133–1.772)^A^	1.593 (1.309–1.808)^A^	1.652 (1.155–2.361)^A^
Cord protrusion	Ref	1.242 (0.737–2.091)	1.560 (1.095–2.223)	1.508 (0.664–3.425)
Cord entanglement	Ref	0.910 (0.850–0.974)^B^	1.283 (1.227–1.342)^A^	1.098 (0.984–1.226)
Cord torsion	Ref	0.980 (0.905–1.062)	1.158 (1.096–1.224)^A^	1.061 (0.927–1.214)
Rupture of uterus	Ref	0.471 (0.361–0.615)^B^	1.172 (1.029–1.335)^A^	0.833 (0.582–1.192)
Postpartum embolism	Ref	1.548 (0.653–3.668)	1.770 (0.957–3.275)	3.343 (1.191–9.385)^A^
Postpartum thrombosis	Ref	1.07 (0.707–1.619)	1.807 (1.409–2.319)^A^	1.804 (1.030–3.160)^A^
PTB^A^	Ref^A^	2.204 (2.064–2.353^A^)	1.317 (1.243–1.396)^A^	2.765 (2.496–3.064)^A^
Macrosomia	Ref	0.923 (0.831–1.026)	1.007 0.934–1.085)	1.000 (0.837–1.194)
LBW^A^	Ref^A^	3.439 (2.620–4.514)^A^	1.442 (1.082–1.923)^A^	4.241 (2.833–6.351)^A^
Neonatal asphyxia	Ref	1.745 (1.382–2.204)^A^	1.025 (0.826–1.272)	2.361 (1.671–3.336)^A^
PPH	Ref	1.241 (1.122–1.373)^A^	1.036 (0.956–1.122)	1.075 (0.893–1.294)
CS^A^	Ref^A^	1.752 (1.680–1.827)^A^	1.320 (1.281–1.361)^A^	2.421 (2.244–2.612)^A^
Intrauterine distress	Ref	1.096 (1.013–1.186)^A^	0.895 (0.841–0.953)^B^	0.928 (0.802–1.075)
FGR^A^	Ref^A^	8.559 (6.004–12.202)^A^	1.800 (1.135–2.856)^A^	9.562 (5.785–15.804)^A^
SGA^A^	Ref^A^	4.041 (2.861–5.707)^A^	1.847 (1.297–2.630)^A^	7.712 (5.052–11.771)^A^
Anemia	Ref^B^	0.851 (0.790–0.916)^B^	0.777 (0.735–0.821)^B^	0.666 (0.577–0.768)^B^
Polyhydramnios	Ref	0.967 (0.807–1.160)	1.630 (1.462–1.818)^A^	1.356 (1.035–1.776)^A^
Oligohydramnios	Ref	1.031 (0.924–1.150)	0.732 (0.667–0.802)^B^	0.835 (0.678–1.030)
Amniotic fluid contamination	Ref	0.957 (0.742–1.233)	0.988 (0.822–1.187)	0.891 (0.564–1.409)

*Note:* Odds ratios (ORs) and 95% confidence intervals (CIs) were derived from unadjusted analyses. Uppercase letter “A” indicates significantly increased risk in twin versus singleton pregnancies (*p* < 0.05); “B” indicates significantly decreased risk.

Abbreviations: CS, cesarean section; FGR, fetal growth restriction; OR, odds ratio (unadjusted, crude OR); LBW, low birth weight; PPH, postpartum hemorrhage; PROM, premature rupture of membranes; PTB, preterm births; SGA, small for gestational age; UA, due to the case being 0, the OR value was unavailable.

### 3.4. The Differences in Adverse Outcomes Among Different HDP/GDM Subgroups in the Twin Pregnancies

Among all twin pregnancies (*n* = 2803), no differences in adverse outcome risks were observed across the four groups, except for PTB, CS, and amniotic fluid contamination (Table S11). The odds of PTB in the HDP, GDM, and HDP‐GDM groups were 1.31 times, 1.18 times, and 1.39 times that of the non–HDP‐GDM group, respectively. The odds of amniotic fluid abnormality in the HDP‐GDM group were 5.9 times that of the control group (OR = 5.907) (Table 5).

**Table 5:** The difference in twin outcomes between those affected solely by HDP or GDM, and those affected simultaneously by HDP and GDM (with risk in non–HDP‐GDM twin pregnancies as the reference).

Variables	Groups			
	Non–HDP‐GDM group	HDP group OR (95% CI)	GDM group OR (95% CI)	HDP‐GDM group OR (95% CI)
Placenta previa	Ref	1.179 (0.694–2.003)	1.042 (0.584–1.860)	1.262 (0.534–2.981)
PROM	Ref	0.768 (0.559–1.054)	0.876 (0.637–1.205)	0.677 (0.384–1.195)
Placental abruption	Ref	0.832 (0.362–1.910)	1.073 (0.487–2.364)	2.050 (0.781–5.376)
Placental implantation	Ref	1.609 (0.806–3.213)	1.047 (0.451–2.427)	0.897 (0.211–3.817)
Cord protrusion	Ref	UA	1.947 (0.355–10.662)	UA
Cord entanglement	Ref	1.321 (0.869–2.010)	1.151 (0.728–1.821)	1.401 (0.711–2.761)
Cord torsion	Ref	1.086 (0.659–1.792)	1.228 (0.744–2.029)	0.513 (0.159–1.652)
Rupture of uterus	Ref	1.080 (0.394–2.964)	1.960 (0.833–4.609)	2.215 (0.638–7.690)
Postpartum embolism	Ref	0.863 (0.096–7.742)	0.971 (0.108–8.710)	5.907 (1.073–32.523)
Postpartum thrombosis	Ref	0.344 (0.044–2.695)	1.953 (0.664–5.743)	2.354 (0.511–10.848)
PTB	Ref	1.312 (1.059–1.626)^A^	1.287 (1.029–1.611)^A^	1.398 (0.967–2.022)^A^
Macrosomia	Ref	UA	UA	UA
LBW	Ref	0.725 (0.246–2.141)	0.611 (0.180–2.073)	0.612 (0.081–4.605)
Neonatal asphyxia	Ref	0.671 (0.338–1.331)	0.834 (0.431–1.613)	0.451 (0.109–1.870)
PPH	Ref	1.387 (0.898–2.143)	1.077 (0.657–1.767)	1.080 (0.488–2.386)
CS	Ref	1.990 (1.048–3.779)	1.765 (0.929–3.354)	2.162 (0.673–6.943)
Intrauterine distress	Ref	1.565 (0.708–3.459)	2.368 (1.149–4.882)^A^	2.373 (0.800–7.038)
FGR	Ref	1.856 (0.782–4.404)	0.775 (0.223–2.690)	1.565 (0.354–6.910)
SGA	Ref	2.030 (0.795–5.183)	1.298 (0.416–4.043)	0.973 (0.126–7.537)
Anemia	Ref	1.179 (0.915–1.520)	0.828 (0.618–1.108)	0.845 (0.528–1.352)
Polyhydramnios	Ref	1.185 (0.641–2.192)	1.735 (0.987–3.051)	0.852 (0.260–2.784)
Oligohydramnios	Ref	0.766 (0.258–2.273)	1.730 (0.752–4.028)	0.646 (0.086–4.877)
Amniotic fluid contamination	Ref	UA	UA	5.907 (1.073–32.523)^A^

*Note:* Odds ratios (ORs) and 95% confidence intervals (CIs) were derived from unadjusted analyses. Uppercase later “A” indicates significantly increased risk in twin versus singleton pregnancies (*p* < 0.05).

Abbreviations: CS, cesarean section; FGR, fetal growth restriction; LBW, low birth weight; OR, odds ratio (unadjusted, crude OR); PPH, postpartum hemorrhage; PROM, premature rupture of membranes; PTB, preterm births; SGA, small for gestational age; UA, due to the case being 0, the OR value was unavailable.

## 4. Discussion

### 4.1. Main Findings

The primary findings of this study are summarized as follows: (1) Significant differences were observed in maternal and neonatal complications between singleton and twin pregnancies (e.g., GDM and HDP), and this discrepancy remained consistent across term and preterm infant subgroups. (2) The maternal and perinatal risk of twin pregnancies with GDM or HDP was not significantly increased as much as expected. There were 3, 6, 8, and 15 complications with no significant difference in the risk of perinatal complications between singleton and twin pregnancies in the non–HDP‐GDM group, HDP group, GDM group, and HDP‐GDM group, respectively. This suggests that the risk increments associated with HDP and GDM in singleton pregnancies were not equivalently amplified in twin pregnancies. (3) In singleton pregnancies, compared with the non–HDP‐GDM group, the combined effect of HDP and GDM manifested as either synergistic enhancement (observed in 11 variables) or attenuated cancelation (observed in three variables), depending on whether the individual effects of HDP and GDM were concordant. (4) However, no statistically significant differences in the incidence of various comorbidities were observed among the four twin pregnancy groups, except for PTB and CS.

### 4.2. Strengths and Limitations

Given that it is clinically unfeasible to withhold treatment from pregnant women with HDP and GDM, studies on pregnant women with HDP/GDM are mainly retrospective, and sufficient clinical cases are required to accurately reflect clinical phenomena and trends. The main strengths of our study include the large sample size covering both singleton and twin pregnancies. Second, it was conducted against the social and clinical backdrop of a significant increase in ART utilization and twin birth rates, and it represents the first combined analysis of the clinical effects of GDM and HDP. However, several limitations warrant attention when interpreting these findings. First, as a single tertiary hospital‐based study, pregnancies enrolled in our center—even those in the non–HDP‐GDM group—were more prone to high‐risk characteristics. Second, as a retrospective real‐world study, we were unable to capture and eliminate all potential confounding variables through randomization methods [19]. The key confounder in this study is the marked disparities in maternal age and assisted reproductive technology use across singleton and twin pregnancies. In addition, all reported ORs are crude values, potentially limiting the reliability of our findings. Third, the enrolled cases were primarily from Wuhan and Hubei regions, which limits regional representativeness; thus, we cannot confirm whether the results are consistent among pregnant women of different ethnicities or regions. Fourth, we could not implement a prospective study, and no basic research has elucidated the mechanism underlying our findings.

### 4.3. Interpretation

As a real‐world study, the results of this research are statistical findings based on clinical data, which only reflect clinical phenomena and trends. The potential mechanisms underlying these results involve multiple aspects. First, the proportion of ART in the twin population is relatively high. Studies have shown that ART twin pregnancies share common risk factors for HDP and GDM, such as advanced maternal age and primiparity [20, 21]. Therefore, the higher proportion of ART may partially account for the elevated incidence of HDP, GDM, and other perinatal complications in twin pregnancies [22]. Second, additional contributing factors to the heightened risk of HDP and GDM include, but are not limited to, increased insulin resistance, greater placental mass, enhanced immunogenicity, and elevated intra‐abdominal pressure (IAP). Such potential mechanisms are also supported by relevant research findings [23]. Although existing literature on the incidence of GDM in twin pregnancies presents conflicting findings, its prevalence is undeniably nonnegligible [12, 24, 25]. Consistent with prior studies [6], we similarly observed that singleton pregnancies with concurrent GDM exhibit an increased risk of HDP compared with those without GDM—a pattern that also holds true in twin pregnancies.

The third potential mechanism is the doubled fetal count and incomplete structural and functional development of the placenta [26]. The growing trend toward single embryo transfer further confirms that perinatal outcomes are closely associated with fetal count [27, 28]. Furthermore, in singleton pregnancies, both HDP and GDM have been linked to an elevated risk of adverse maternal and perinatal outcomes; however, the impact of HDP and GDM in twin pregnancies—particularly their coexistence—remains unclear [14, 29]. Consequently, twin pregnancies, characterized by inherent high risk and a significantly heightened risk of HDP and GDM, have become a key focus of current research [30]. The impact of HDP and GDM on fetal weight has been the most extensively studied aspect. Evidence indicates that compared with singleton pregnancies, the effect of HDP and GDM on fetal growth is less pronounced in twin pregnancies [31]. The discrepancy in fetal weight and growth rates may be attributed to the reduced growth potential during the third trimester in twin pregnancies, thereby reducing the likelihood of macrosomia development [32, 33] .

Our study corroborates the protective hypothesis: in twin pregnancies complicated by GDM, the risk of pregnancy complications does not increase significantly, whereas a reduced risk of pregnancy complications was observed in HDP‐complicated twin pregnancies. The combined impact of HDP and GDM on twin pregnancies remains limited. This study′s data demonstrated that, except for PTB and CS, the incidences of other comorbidities did not differ significantly among the four twin pregnancy groups. This finding may be attributable to the intrinsically elevated baseline risk of twin gestation, which could mask the additional adverse risks conferred by HDP and GDM. Alternatively, HDP and GDM may exhibit fundamentally distinct pathophysiological mechanisms in twin pregnancies compared with singleton pregnancies. Nevertheless, additional mechanistic investigations are required to clarify whether the inherent heightened risk in twins is associated with more frequent maternal‐fetal interactions. Equally urgent is exploring how the adverse effects of HDP and GDM in singleton pregnancies might function as protective mechanisms in twin pregnancies.

## 5. Conclusion

Notably, the greatest strength of this study lies in challenging the traditional perception of additive risks among GDM, HDP, and twin pregnancies. It provides evidence challenging the traditional perception. It suggests that the associated risk from the co‐occurrence of GDM and HDP is milder in twin than in singleton pregnancies. Thus, the findings indicate that twin pregnancies cannot be fully managed using the same protocols as singleton pregnancies. It is imperative to conduct further stratified research and targeted management. Prevention and control of GDM, especially GDM comorbid with HDP, help reduce pregnancy‐related risks. The data obtained from this study will help provide more personalized guidance for twin pregnancies, optimize prenatal care, and offer evidence for broader public health strategies, thereby achieving substantive outcomes. This study was a single‐center real‐world investigation, and the generalizability of its findings remains to be further validated by large‐sample and multiethnic cohort studies. Future studies should conduct multicenter validation trials, explore the physiological mechanisms underlying the attenuation of this protective effect, and establish evidence‐based clinical management protocols specifically for twin pregnancies.

## Author Contributions

P.G. and J.B. participated in designing the study. J.B. organized and facilitated the data collection. P.G. analyzed the data and provided expertise and supervision in data analyses. All authors participated in the discussion of the findings and critical.

## Funding

No funding was received for this manuscript.

## Ethics Statement

The observational research protocol for this study was approved by the Medical Ethics Committee of Maternal and Child Health Hospital of Hubei Province. The studies were conducted in accordance with the local legislation and institutional requirements. The ethics committee/institutional review board waived the requirement of written informed consent for participation from the participants or the participants’ legal guardians because it was a retrospective study. We only extracted relevant clinical information, did not affect the treatment and health of patients, and the patients′ privacy was protected.

## Conflicts of Interest

The authors declare no conflicts of interest.

## Supporting Information

Additional supporting information can be found online in the Supporting Information section.

## Supporting information


**Supporting Information 1** This study incorporates a total of 11 supporting tables that complement and reinforce the research findings. Tables S1, S2, S3, S4, S5, and S6 serve as supplements to Table 1. Among these, Tables S1, S2, and S3 analyze the differences in the incidence of pregnancy complications and pregnancy outcomes between singleton and twin pregnancies under preterm and term conditions: Table S1: Comparison of pregnancy complications and outcomes between term singleton and twin pregnancies. Table S2: Comparison of pregnancy comorbidities and outcomes between preterm singleton and twin pregnancies. Table S3: Risk of complications and adverse outcomes in twin pregnancies (with singleton pregnancies as the reference). Tables S4 and S5 primarily present the differences in the overall and subtype‐specific incidence rates of HDP and GDM between singleton and twin pregnancies: Table S4: Differences in HDP subgroups between singleton and twin pregnancies. Table S5: Differences in GDM subgroups between singleton and twin pregnancies. Tables S6, S7, S8, and S9 supplement Table 3, with detailed comparisons of singleton and twin pregnancies conducted across different groups: Table S6: Comparison of perinatal complications between singleton and twin pregnancies in the non–HDP‐GDM group. Table S7: Comparison of perinatal complications between singleton and twin pregnancies in the HDP group. Table S8: Comparison of perinatal complications between singleton and twin pregnancies in the GDM group. Table S9: Comparison of perinatal complications between singleton and twin pregnancies in the HDP‐GDM group. Table S10 supplements Table 4, comparing the differences in complication incidence rates between different HDP/GDM subgroups in singleton pregnancies: Table S10: Comparison of perinatal complications between different HDP/GDM groups in singleton pregnancies. Table S11 supplements Table 5, comparing the differences in complication incidence rates between different HDP/GDM subgroups in singleton pregnancies: Table S11: Comparison of perinatal complications between different HDP/GDM groups in twin pregnancies.

## Data Availability

Personal data are not available for public sharing. The data presented in this study are available on request from the corresponding author.
