# Supplementary material for: Attenuated Impact of Gestational Diabetes Mellitus (GDM) With Hypertensive Disorders of Pregnancy (HDP) on Adverse Pregnancy Outcomes in Twin Versus Singleton Gestations: A Single‐Center Real‐World Study
Source: J Diabetes Res. 2026 Aug 16;2026:3096949. doi: 10.1155/jdr/3096949 (PMC13478717; doi:10.1155/jdr/3096949)
Supplement: Supplementary file 1 [file JDR-2026-3096949-s001.docx]

**Table S1. The comparison of pregnant complications and outcomes between term singleton and twin pregnancies.**

| **Variables** | **Total term pregnancies (n=120597, %)** | **Groups** | | | | **OR (95%CI)**  **(twin vs singleton)** |
| --- | --- | --- | --- | --- | --- | --- |
|  |  | **Singleton pregnancies (n=119636, %)** | | **Twin pregnancies （n=961, %）** | ***P* value** |  |
| ART |  |  | |  |  |  |
| yes | 3211 (2.7) | 2963 (2.5) | | 248 (25.8) | <0.001 | 13.696 (11.800-15.897) |
| no | 117386 (97.3) | 116673 (97.5) | | 713 (74.2) |  |  |
| HDP |  |  | |  |  |  |
| yes | 11563 (9.6) | 11368 (9.5) | | 195 (20.3) | <0.001 | 2.424 (2.069-2.841) |
| no | 109034 (90.4) | 108268 (90.5) | | 766 (79.7) |  |  |
| GDM |  |  | |  |  |  |
| yes | 21575 (17.9) | 21395 (17.9) | | 180 (18.7) | 0.495 | 1.058 (0.899-1.245) |
| no | 99022 (82.1) | 98241 (82.1) | | 781 (81.3) |  |  |
| Placenta previa | |  | |  |  |  |
| yes | 1974 (1.6) | 1951 (1.6) | | 23 (2.4) | 0.064 | 1.479 (0.976-2.242) |
| no | 118623 (98.4) | 117685 (98.4) | | 938 (97.6) |  |  |
| PROM |  |  | |  |  |  |
| yes | 20367 (16.9) | 20304 (17.0) | | 63 (6.6) | <0.001 | 0.343 (0.266-0.443) |
| no | 100230 (83.1) | 105166 (83.0) | | 898 (93.4) |  |  |
| Placental abruption | |  | |  |  |  |
| yes | 628 (0.5) | 615 (0.5) | | 13 (1.4) | <0.001 | 2.654 (1.527-4.614) |
| no | 119969 (99.5) | 119021 (99.5) | | 948 (98.6) |  |  |
| Placental implantation | | |  |  |  |  |
| yes | 646 (0.5) | 636 (0.5) | | 10 (1.0) | 0.031 | 1.967 (1.050-3.686) |
| no | 119951 (99.5) | 119000 (99.5) | | 951 (99.0) |  |  |
| Cord protrusion | |  | |  |  |  |
| yes | 156 (0.1) | 156 (0.1) | | 0 (0) | 0.416 | UA |
| no | 120441 (99.9) | 119480 (99.9) | | 961 (100) |  |  |
| Cord entanglement | |  | |  |  |  |
| yes | 13971 (11.6) | 13922 (11.6) | | 49 (5.1) | <0.001 | 0.408 (0.306-0.544) |
| no | 106626 (88.4) | 105714 (88.5) | | 912 (94.6) |  |  |
| Cord torsion | |  | |  |  |  |
| yes | 8900 (7.4) | 8867 (7.4) | | 33 (3.4) | <0.001 | 0.444 (0.314-0.629) |
| no | 111697 (92.6) | 110769 (92.6) | | 928 (96.6) |  |  |
| Rupture of uterus | |  | |  |  |  |
| yes | 1290 (1.1) | 1280 (1.1) | | 10 (1.0) | 0.930 | 0.972 (0.520-1.817) |
| no | 119307 (98.9) | 118356 (98.9) | | 951 (99.0) |  |  |
| Postpartum embolism | | |  |  |  |  |
| yes | 52 (0) | 50 (0) | | 2 (0.2) | 0.026 | 4.988 (1.212-20.528) |
| no | 120545 (100) | 119586 (100) | | 959 (99.8) |  |  |
| Postpartum thrombosis | | |  |  |  |  |
| yes | 287 (0.2) | 283 (0.2) | | 4 (0.4) | 0.298 | 1.763 (0.656-4.739) |
| no | 120310 (99.8) | 119353 (99.8) | | 957 (99.6) |  |  |
| Macrosomia | |  | |  |  |  |
| yes | 5431 (4.5) | 5429 (4.5) | | 2 (0.1) | <0.001 | 0.044 (0.011-0.176) |
| no | 115166 (95.5) | 114207 (95.5) | | 959 (99.8) |  |  |
| LBW |  |  | |  |  |  |
| yes | 300 (0.2) | 287 (0.2) | | 13 (1.4) | <0.001 | 5.703 (3.259-9.978) |
| no | 120297 (99.8) | 119349 (99.8) | | 948 (98.6) |  |  |
| Neonatal asphyxia | |  | |  |  |  |
| yes | 376 (0.3) | 369 (0.3) | | 7 (0.7) | 0.032 | 2.372 (1.120-5.023) |
| no | 120221 (99.7) | 119267 (99.7) | | 954 (99.3) |  |  |
| PPH |  |  | |  |  |  |
| yes | 4581 (3.8) | 4523 (3.8) | | 58 (6.0) | <0.001 | 1.635 (1.251-2.135) |
| no | 116016 (96.2) | 115113 (96.2) | | 903 (94.0) |  |  |
| CS |  |  | |  |  |  |
| yes | 56172 (46.6) | 55236 (46.2) | | 936 (97.4) | <0.001 | 43.652 (29.338-64.948) |
| no | 64425 (53.4) | 64400 (53.8) | | 25 (2.6) |  |  |
| Intrauterine distress | |  | |  |  |  |
| yes | 8176 (6.8) | 8169 (6.8) | | 7 (0.7) | <0.001 | 0.100 (0.048-0.211) |
| no | 112421 (93.2) | 111467 (93.2) | | 954 (99.3) |  |  |
| FGR |  |  | |  |  |  |
| yes | 75 (0.1) | 69 (0.1) | | 6 (0.6) | <0.001 | 10.887 (4.716-25.134) |
| no | 120522 (99.9) | 119567 (99.9) | | 955 (99.4) |  |  |
| SGA |  |  | |  |  |  |
| yes | 123 (0.1) | 118 (0.1) | | 5 (0.5) | <0.001 | 5.297 (2.160-12.993) |
| no | 120474 (99.9) | 119518 (99.9) | | 956 (99.5) |  |  |
| Anemia |  |  | |  |  |  |
| yes | 11140 (9.2) | 10977 (9.2) | | 163 (17.0) | <0.001 | 2.022 (1.707-2.396) |
| no | 109457 (90.8) | 108659 (90.8) | | 798 (83.0) |  |  |
| Polyhydramnios | |  | |  |  |  |
| yes | 1756 (1.5) | 1742 (1.5) | | 14 (1.5) | 0.998 | 1.001 (0.589-1.699) |
| no | 118841 (98.5) | 117894 (98.5) | | 947 (98.5) |  |  |
| Oligohydramnios | |  | |  |  |  |
| yes | 3845 (3.2) | 3836 (3.2) | | 9 (0.9) | <0.001 | 0.285 (0.148-0.551) |
| no | 116752 (96.8) | 115800 (96.8) | | 952 (99.1) |  |  |
| Amniotic fluid contamination | | |  |  |  |  |
| yes | 872 (0.7) | 871 (0.7) | | 1 (0.1) | <0.001 | 0.142 (0.020-1.010) |
| no | 119725 (99.3) | 118765 (99.3) | | 960 (99.9) |  |  |
| **Abbreviations:** ART: assisted reproductive technology, HDP: hypertensive disorders of pregnancy, GDM: gestational diabetes mellitus, PROM: premature rupture of membranes, PTB: preterm births, LBW: low birth weight, PPH: postpartum hemorrhage, CS: cesarean section, FGR: fetal growth restriction, SGA: Small for gestational age, UA:Due to the case being 0, the value was unavailable. | | | | | | |

**Table S2**. **The comparison of pregnant comorbidities and outcomes between preterm singleton and twin pregnancies.**

| **Variables** | **Total preterm pregnancies (n=10799, %)** | **Groups** | | | **OR (95%CI)**  **(twin vs singleton)** |
| --- | --- | --- | --- | --- | --- |
|  |  | **Singleton pregnancies (n=8957, %)** | **Twin pregnancies （n=1842, %）** | ***P* value** |  |
| ART |  |  |  |  |  |
| yes | 736 (6.8) | 290 (3.2) | 446 (24.2) | <0.001 | 9.548 (8.150-11.186) |
| no | 10063 (93.2) | 8776 (96.8) | 1396 (75.8) |  |  |
| HDP |  |  |  |  |  |
| yes | 2137 (19.8) | 1688 (18.8) | 449 (24.4) | <0.001 | 1.388 (1.233-1.563) |
| no | 8662 (80.2) | 7269 (81.2) | 1393 (75.6) |  |  |
| GDM |  |  |  |  |  |
| yes | 2444 (22.6) | 2035 (22.7) | 409 (22.2) | 0.630 | 0.971 (0.861-1.095) |
| no | 8355 (77.4) | 6922 (77.3) | 1433 (77.8) |  |  |
| Placenta previa | |  |  |  |  |
| yes | 1009 (9.3) | 936 (10.4) | 73 (4.0) | <0.001 | 0.354 (0.277-0.451) |
| no | 9790 (90.7) | 8021 (89.6) | 1769 (96.0) |  |  |
| PROM |  |  |  |  |  |
| yes | 3411 (31.6) | 3123 (34.9) | 288 (15.6) | <0.001 | 0.346 (0.303-0.395) |
| no | 7388 (68.4) | 5834 (65.1) | 1554 (84.4) |  |  |
| Placental abruption | |  |  |  |  |
| yes | 431 (4.0) | 395 (4.4) | 36 (2.0) | <0.001 | 0.432 (0.306-0.610) |
| no | 10368 (96.0) | 8562 (95.6) | 1806 (98.0) |  |  |
| Placental implantation | |  |  |  |  |
| yes | 320 (3.0) | 283 (3.2) | 37 (2.0) | 0.008 | 0.628 (0.444-0.888) |
| no | 10479 (97.0) | 8674 (96.8) | 1805 (98.0) |  |  |
| Cord protrusion | |  |  |  |  |
| yes | 36 (0.3) | 30 (0.3) | 6 (0.3) | 0.950 | 0.972 (0.404-2.340) |
| no | 10763 (99.7) | 8927 (99.7) | 1836 (99.7) |  |  |
| Cord entanglement | |  |  |  |  |
| yes | 1006 (9.3) | 903 (10.1) | 103 (5.6) | <0.001 | 0.528 (0.428-0.652) |
| no | 9793 (90.7) | 8054 (89.9) | 1739 (94.4) |  |  |
| Cord torsion | |  |  |  |  |
| yes | 791 (7.3) | 712 (7.9) | 79 (4.3) | <0.001 | 0.519 (0.409-0.658) |
| no | 10008 (92.7) | 8245 (92.1) | 1763 (95.7) |  |  |
| Rupture of uterus | |  |  |  |  |
| yes | 265 (2.5) | 243 (2.7) | 22 (1.2) | <0.001 | 0.433 (0.279-0.673) |
| no | 10534 (97.5) | 8714 (97.3) | 1820 (98.8) |  |  |
| Postpartum embolism | |  |  |  |  |
| yes | 17 (0.2) | 11 (0.1) | 6 (0.3) | 0.055 | 2.658 (0.982-7.196) |
| no | 10782 (99.8) | 8946 (99.9) | 1836 (99.7) |  |  |
| Postpartum thrombosis | |  |  |  |  |
| yes | 78 (0.7) | 64 (0.7) | 14 (0.8) | 0.834 | 1.064 (0.596-1.902) |
| no | 10721 (99.3) | 8893 (99.3) | 1828 (99.2) |  |  |
| Macrosomia | |  |  |  |  |
| yes | 15 (0.1) | 15 (0.2) | 0 (0) | 0.091 | UA |
| no | 10784 (99.9) | 8942 (99.8) | 1842 (95.5) |  |  |
| LBW |  |  |  |  |  |
| yes | 84 (0.8) | 70 (0.8) | 14 (0.8) | 0.924 | 0.972 (0.547-1.730) |
| no | 10715 (99.2) | 8887 (99.2) | 1828 (99.2) |  |  |
| Neonatal asphyxia | |  |  |  |  |
| yes | 379 (3.5) | 312 (3.5) | 67 (3.6) | 0.744 | 1.046 (0.799-1.368) |
| no | 10420 (96.5) | 8645 (96.5) | 1775 (96.4) |  |  |
| PPH |  |  |  |  |  |
| yes | 343 (3.2) | 267 (3.0) | 76 (4.1) | 0.011 | 1.401 (1.080-1.816) |
| no | 10456 (96.8) | 8690 (97.0) | 1766 (95.9) |  |  |
| CS |  |  |  |  |  |
| yes | 7745 (71.7) | 5977 (66.7) | 1768 (96.0) | <0.001 | 11.912 (9.401-15.093) |
| no | 3054 (28.3) | 2980 (33.3) | 74 (4.0) |  |  |
| Intrauterine distress | |  |  |  |  |
| yes | 635 (5.9) | 597 (6.7) | 38 (2.1) | <0.001 | 0.295 (0.212-0.411) |
| no | 10164 (94.1) | 8360 (93.3) | 1804 (97.9) |  |  |
| FGR |  |  |  |  |  |
| yes | 121 (1.1) | 99 (1.1) | 22 (1.2) | 0.741 | 1.082 (0.680-1.721) |
| no | 10678 (98.9) | 8858 (98.9) | 1820 (98.8) |  |  |
| SGA |  |  |  |  |  |
| yes | 126 (1.2) | 107 (1.2) | 19 (1.0) | 0.553 | 0.862 (0.528-1.408) |
| no | 10673 (98.8) | 8850 (98.8) | 1823 (99.0) |  |  |
| Anemia |  |  |  |  |  |
| yes | 1205 (11.2) | 887 (9.9) | 318 (17.3) | <0.001 | 1.898 (1.652-2.182) |
| no | 9594 (80.8) | 8070 (90.1) | 1524 (82.7) |  |  |
| Polyhydramnios | |  |  |  |  |
| yes | 231 (2.1) | 169 (1.9) | 62 (3.4) | <0.001 | 1.811 (1.348-2.434) |
| no | 10568 (97.9) | 8788 (98.1) | 1780 (96.6) |  |  |
| Oligohydramnios | |  |  |  |  |
| yes | 607 (5.6) | 585 (6.5) | 22 (3.6) | <0.001 | 0.173 (0.113-0.266) |
| no | 10192 (94.4) | 8372 (93.5) | 1820 (98.8) |  |  |
| Amniotic fluid contamination | |  |  |  |  |
| yes | 16 (0.1) | 11 (0.1) | 5 (0.3) | 0.172 | 2.214 (0.768-6.378) |
| no | 10783 (99.9) | 8946 (99.9) | 1837 (99.7) |  |  |
| **Abbreviations:** ART: assisted reproductive technology, HDP: hypertensive disorders of pregnancy, GDM: gestational diabetes mellitus, PROM: premature rupture of membranes, PTB: preterm births, LBW: low birth weight, PPH: postpartum hemorrhage, CS: cesarean section, FGR: fetal growth restriction, SGA: Small for gestational age. UA: Due to the case being 0, the value was unavailable. | | | | | |

**Table S3**. **The risk of complications and adverse outcomes twin pregnancies** (with risk in singleton as the reference)**.**

| **Variables** | **Risk in pregnancies (twin vs singleton), OR (95%CI)** | | |
| --- | --- | --- | --- |
|  | **Total pregnancies** | **Term pregnancies** | **Preterm pregnancies** |
| ART | 12.549 (11.444-13.760) | 13.696 (11.800-15.897) | 9.548 (8.150-11.186) |
| HDP | 2.640 (2.413-2.888) | 2.424 (2.069-2.841) | 1.388 (1.233-1.563) |
| GDM | 1.194 (1.089-1.309) | 1.058 (0.899-1.245) | 0.971 (0.861-1.095) |
| Placenta previa | 1.544 (1.256-1.899) | 1.479 (0.976-2.242) | 0.354 (0.277-0.451) |
| PROM | 0.643 (0.564-0.719) | 0.343 (0.266-0.443) | 0.346 (0.303-0.395) |
| Placental abruption | 2.248 (1.683-3.001) | 2.654 (1.527-4.614) | 0.432 (0.306-0.610) |
| Placental implantation | 2.369 (1.763-3.184) | 1.967 (1.050-3.686) | 0.628 (0.444-0.888) |
| Cord protrusion | 1.481 (0.656-3.342) | UA | 0.972 (0.404-2.340) |
| Cord entanglement | 0.440 (0.373-0.519) | 0.408 (0.306-0.544) | 0.528 (0.428-0.652) |
| Cord torsion | 0.517 (0.428-0.625) | 0.444 (0.314-0.629) | 0.519 (0.409-0.658) |
| Rupture of uterus | 0.964 (0.678-1.370) | 0.972 (0.520-1.817) | 0.433 (0.279-0.673) |
| Postpartum embolism | 6.031 (2.883-12.615) | 4.988 (1.212-20.528) | 2.658 (0.982-7.196) |
| Postpartum thrombosis | 2.389 (1.485-3.842) | 1.763 (0.656-4.739) | 1.064 (0.596-1.902) |
| PTB | 25.602 (23.612-27.759) | UA | UA |
| Macrosomia | 0.016 (0.004-0.065) | 0.044 (0.011-0.176) | UA |
| LBW | 3.494 (2.358-5.176) | 5.703 (3.259-9.978) | 0.972 (0.547-1.730) |
| Neonatal asphyxia | 5.093 (3.995-6.493) | 2.372 (1.120-5.023) | 1.046 (0.799-1.368) |
| PPH | 1.298 (1.088-1.547) | 1.635 (1.251-2.135) | 1.401 (1.080-1.816) |
| CS | 30.065 (24.594-36.753) | 43.652 (29.338-64.948) | 11.912 (9.401-15.093) |
| Intrauterine distress | 0.223 (0.166-0.300) | 0.100 (0.048-0.211) | 0.295 (0.212-0.411) |
| FGR | 7.713 (5.161-11.528) | 10.887 (4.716-25.134) | 1.082 (0.680-1.721) |
| SGA | 4.927 (3.229-7.518) | 5.297 (2.160-12.993) | 0.862 (0.528-1.408) |
| Anemia | 2.038 (1.844-2.252) | 2.022 (1.707-2.396) | 1.898 (1.652-2.182) |
| Polyhydramnios | 1.847 (1.464-2.331) | 1.001 (0.589-1.699) | 1.811 (1.348-2.434) |
| Oligohydramnios | 0.314 (0.220-0.448) | 0.285 (0.148-0.551) | 0.173 (0.113-0.266) |
| Amniotic fluid contamination | 0.311 (0.139-0.694) | 0.142 (0.020-1.010) | 2.214 (0.768-6.378) |
| **Abbreviations:** ART: assisted reproductive technology, HDP: hypertensive disorders of pregnancy, GDM: gestational diabetes mellitus, PROM: premature rupture of membranes, PTB: preterm births, LBW: low birth weight, PPH: postpartum hemorrhage, CS: cesarean section, FGR: fetal growth restriction, SGA: Small for gestational age. UA: Due to the case being 0, the value was unavailable. | | | |

**Table S4**. **Differences in HDP subgroups between singleton and twin pregnancies.**

| **Variables** | **Pregnancies with HDP ^**^(n=13700, %)** | **Groups** | | | |  |
| --- | --- | --- | --- | --- | --- | --- |
|  |  | **Singleton pregnancies (n=13056, %)** | **Twin pregnancies (n=644, %)** | ***P* value** | **OR (95%CI)**  **(twin vs singleton)** |  |
| Chronic hypertension | 542 (0.4) | 529 (0.4) | 13 (0.5) | 0.668 | 1.128 (0.650-1.958) |  |
| GH | 9807 (7.4) | 9488 (7.4) | 319 (11.4) | <0.001 | 1.612 (1.432-1.815) |  |
| Mild PE | 1649 (1.3) | 1485 (1.2) | 164 (5.9) | <0.001 | 5.319 (4.506-6.279) |  |
| Severe PE and eclampsia* | 1702 (1.3) | 1554 (1.2) | 148 (5.3) | <0.001 | 4.557 (3.833-5.417) |  |
| *: There were 6 and 1 cases with eclampsia in singleton group and twin group, respectively.  **：This column shows overall data of all HDP cases, covering singleton and twin pregnancies.  **Abbreviations:** HDP: hypertensive disorders of pregnancy, GH: gestational hypertension, PE: preeclampsia. | | | | | | |

**Table S5. Differences in GDM subgroups between singleton and twin pregnancies.**

| **Variables** | **Pregnancies with GDM (n=24019, %)** | **Groups** | |  |
| --- | --- | --- | --- | --- |
|  |  | **Singleton pregnancies (n=23430, %)** | **Twin pregnancies (n=589, %)** | ***P* value** |
| Nonketotic | 23826 (99.2) | 23243 (99.2) | 583 (99.0) | 0.355 |
| Ketosis | 193 (0.8) | 187(0.8) | 6 (1.0) |  |

**Table S6. Comparison of perinatal complications between singleton and twin pregnancies in Non-HDP-GDM group.**

|  | **Non-HDP-GDM group (n=96905, %)** | **Subgroups** | | |  | |
| --- | --- | --- | --- | --- | --- | --- |
| **Variables** |  | **Singleton pregnancies (n=95188, %)** | **Twin pregnancies (n=1717, %)** | ***P* value** | | **OR (95%CI)**  **(twin vs singleton)** |
| Placenta previa | |  |  |  | |  |
| yes | 2223 (2.3) | 2167 (2.3) | 56 (3.3) | 0.007 | | 1.447 (1.105-1.895) |
| no | 94682 (97.7) | 125706 (97.7) | 1661 (96.7) |  | |  |
| PROM |  |  |  |  | |  |
| yes | 18356 (18.9) | 18125 (19.0) | 231 (13.5) | <0.001 | | 0.661 (0.575-0.760) |
| no | 78549 (81.1) | 77063 (81.0) | 1486 (86.5) |  | |  |
| Placental abruption | |  |  |  | |  |
| yes | 703 (0.7) | 674 (0.7) | 29 (1.7) | <0.001 | | 2.409 (1.656-3.505) |
| no | 96202 (99.3) | 94514 (99.3) | 1688 (98.3) |  | |  |
| Placental implantation | |  |  |  | |  |
| yes | 627 (0.6) | 601 (0.6) | 26 (1.5) | <0.001 | | 2.420 (1.629-3.594) |
| no | 962728 (99.4) | 94587 (99.4) | 1691 (98.5) |  | |  |
| Cord protrusion | |  |  |  | |  |
| yes | 127 (0.1) | 123 (0.1) | 4 (0.2) | 0.239 | | 1.805 (0.666-4.891) |
| no | 96778 (99.9) | 95065 (99.9) | 1713 (99.8) |  | |  |
| Cord entanglement | |  |  |  | |  |
| yes | 10694 (11.0) | 10609 (11.1) | 85 (5.0) | <0.001 | | 0.415 (0.334-0.517) |
| no | 86211 (89.0) | 84579 (88.9) | 1632 (95.0) |  | |  |
| Cord torsion | |  |  |  | |  |
| yes | 6999 (7.2) | 6932 (7.3) | 67 (3.9) | <0.001 | | 0.517 (0.404-0.661) |
| no | 89906 (92.8) | 88256 (92.7) | 1650 (96.1) |  | |  |
| Rupture of uterus | |  |  |  | |  |
| yes | 1164 (1.2) | 1148 (1.2) | 16 (0.9) | 0.301 | | 0.771 (0.469-1.265) |
| no | 95741 (98.8) | 94040 (98.8) | 1701 (99.1) |  | |  |
| Postpartum embolism | |  |  |  | |  |
| yes | 41 (<0.1) | 37 (<0.1) | 4 (0.2) | <0.001 | | 6.005 (2.138-16.866) |
| no | 96864 (100) | 95151 (100) | 1713 (99.7) |  | |  |
| Postpartum thrombosis | |  |  |  | |  |
| yes | 233 (0.2) | 223 (0.2) | 10 (0.6) | 0.004 | | 2.495 (1.322-4.709) |
| no | 96672 (99.8) | 94965 (99.8) | 1707 (99.4) |  | |  |
| PTB |  |  |  |  | |  |
| yes | 6783 (6.0) | 5695 (6.0) | 1088 (63.4) | <0.001 | | 27.182 (24.552-30.093) |
| no | 90122 (91.8) | 89493 (94.0) | 629 (36.6) |  | |  |
| Macrosomia | |  |  |  | |  |
| yes | 4051 (4.2) | 4049 (4.3) | 2 (0.1) | <0.001 | | 0.026 (0.007-0.105) |
| no | 92854 (95.8) | 91139 (95.7) | 1715 (99.9) |  | |  |
| LBW |  |  |  |  | |  |
| yes | 217 (0.2) | 198 (0.2) | 19 (1.1) | <0.001 | | 5.368 (3.345-8.616) |
| no | 96688 (99.8) | 94990 (99.8) | 1698 (98.9) |  | |  |
| Neonatal asphyxia | |  |  |  | |  |
| yes | 512 (0.5) | 461 (0.5) | 51 (3.0) | <0.001 | | 6.290 (4.691-8.434) |
| no | 96393 (99.5) | 94727 (99.5) | 1666 (97.0) |  | |  |
| PPH |  |  |  |  | |  |
| yes | 3535 (3.6) | 3459 (3.6) | 76 (4.4) | 0.083 | | 1.228 (0.973-1.550) |
| no | 93370 (96.4) | 91729 (96.4) | 1641 (95.6) |  | |  |
| CS |  |  |  |  | |  |
| yes | 44396 (48.6) | 42753 (44.9) | 1643 (95.7) | <0.001 | | 27.231 (21.565-34.385) |
| no | 52509 (54.2) | 52435 (55.1) | 74 (4.3) |  | |  |
| Intrauterine distress | |  |  |  | |  |
| yes | 6576 (6.8) | 6556 (6.9) | 20 (1.2) | <0.001 | | 0.159 (0.102-0.248) |
| no | 90329 (93.2) | 88632 (93.1) | 1697 (98.8) |  | |  |
| FGR |  |  |  |  | |  |
| yes | 80 (0.1) | 65 (0.1) | 15 (0.9) | <0.001 | | 12.897 (7.342-22.658) |
| no | 96825 (99.9) | 95123 (99.9) | 1702 (99.1) |  | |  |
| SGA |  |  |  |  | |  |
| yes | 121 (0.1) | 109 (0.1) | 12 (0.7) | <0.001 | | 6.139 (3.376-11.165) |
| no | 96784 (99.9) | 95079 (99.9) | 1705 (99.3) |  | |  |
| Anemia |  |  |  |  | |  |
| yes | 9547 (9.9) | 9251 (9.7) | 296 (17.2) | <0.001 | | 1.935 (1.704-2.197) |
| no | 87358 (90.1) | 85937 (90.3) | 1421 (82.8) |  | |  |
| Polyhydramnios | |  |  |  | |  |
| yes | 1323 (1.4) | 1282 (1.3) | 41 (2.4) | <0.001 | | 1.792 (1.308-2.455) |
| no | 95582 (98.6) | 93906 (98.7) | 1676 (97.6) |  | |  |
| Oligohydramnios | |  |  |  | |  |
| yes | 3437 (3.5) | 3419 (3.6) | 18 (1.0) | <0.001 | | 0.284 (0.179-0.453) |
| no | 93468 (96.5) | 91769 (96.4) | 1699 (99.0) |  | |  |
| Amniotic fluid contamination | |  |  |  | |  |
| yes | 662 (0.7) | 658 (0.7) | 4 (0.2) | <0.001 | | 0.335 (0.125-0.898) |
| no | 96243 (99.3) | 94530 (99.3) | 1713 (99.8) |  | |  |
| **Abbreviations:** PROM: premature rupture of membranes, PTB: preterm births, LBW: low birth weight, PPH: postpartum hemorrhage, CS: cesarean section, FGR: fetal growth restriction, SGA: Small for gestational age. | | | | | | |

**Table S7. Comparison of perinatal complications between singleton and twin pregnancies in HDP group.**

|  | **HDP group (n=10472, %)** | **Subgroups** | | |  |
| --- | --- | --- | --- | --- | --- |
| **Variables** |  | **Singleton pregnancies (n=9975, %)** | **Twin pregnancies (n=497, %)** | ***P* value** | **OR (95%CI)**  **(twin vs singleton)** |
| Placenta previa | |  |  |  |  |
| yes | 189 (1.8) | 170 (1.7) | 19 (3.8) | 0.001 | 2.239 (1.414-3.716) |
| no | 10283 (97.7) | 9805 (98.3) | 478 (96.2) |  |  |
| PROM |  |  |  |  |  |
| yes | 1678 (16.0) | 1625 (16.3) | 53 (10.7) | 0.001 | 0.613 (0.459-0.820) |
| no | 8794 (84.0) | 8350 (83.7) | 444 (89.3) |  |  |
| Placental abruption | |  |  |  |  |
| yes | 127 (1.2) | 120 (1.2) | 7 (1.4) | 0.683 | 1.173 (0.545-2.527) |
| no | 10345 (99.3) | 9855 (98.8) | 490 (98.6) |  |  |
| Placental implantation | |  |  |  |  |
| yes | 101 (1.0) | 89 (0.9) | 12 (2.4) | 0.001 | 2.748 (1.494-5.056) |
| no | 10371 (99.0) | 9886 (99.1) | 485 (97.6) |  |  |
| Cord protrusion | |  |  |  |  |
| yes | 16 (0.2) | 16 (0.2) | 0 (0.0) | 0.372 | UA |
| no | 10456 (99.8) | 9959 (99.8) | 497 (100.0) |  |  |
| Cord entanglement | |  |  |  |  |
| yes | 1054 (10.1) | 1022 (10.2) | 32 (6.4) | 0.006 | 0.603 (0.419-0.868) |
| no | 9418 (89.9) | 8953 (89.8) | 465 (93.6) |  |  |
| Cord torsion |  |  |  |  |  |
| yes | 734 (7.0) | 713 (7.1) | 21 (4.2) | 0.013 | 0.573 (0.368-0.893) |
| no | 9738 (93.0) | 9262 (92.9) | 476 (95.8) |  |  |
| Rupture of uterus | |  |  |  |  |
| yes | 62 (0.6) | 57 (0.6) | 5 (1.0) | 0.218 | 1.768 (0.706-4.431) |
| no | 10410 (99.4) | 9918 (99.4) | 492 (99.0) |  |  |
| Postpartum embolism | |  |  |  |  |
| yes | 7 (0.1) | 6 (0.1) | 1 (0.2) | 0.235 | 3.350 (0.403-27.878) |
| no | 10465 (99.9) | 9969 (99.9) | 496 (99.8) |  |  |
| Postpartum thrombosis | |  |  |  |  |
| yes | 26 (0.2) | 25 (0.3) | 1 (0.2) | 0.829 | 0.802 (0.109-5.934) |
| no | 10446 (99.8) | 9950 (99.7) | 496 (99.8) |  |  |
| PTB |  |  |  |  |  |
| yes | 1572 (15.0) | 1227 (12.3) | 345 (69.4) | <0.001 | 16.182 (13.250-19.764) |
| no | 8900 (85.0) | 88748 (87.7) | 152 (30.6) |  |  |
| Macrosomia |  |  |  |  |  |
| yes | 393 (3.8) | 393 (3.9) | 0 (0.0) | <0.001 | UA |
| no | 10079 (96.2) | 9582 (96.1) | 497 (100.0) |  |  |
| LBW |  |  |  |  |  |
| yes | 75 (0.7) | 71 (0.7) | 4 (0.8) | 0.810 | 1.132 (0.412-3.111) |
| no | 10397 (99.3) | 9904 (99.3) | 493 (99.2) |  |  |
| Neonatal asphyxia | |  |  |  |  |
| yes | 94 (0.9) | 84 (0.8) | 10 (2.0) | <0.001 | 2.418 (1.247-4.687) |
| no | 10378 (99.1) | 9891 (99.2) | 487 (98.0) |  |  |
| PPH |  |  |  |  |  |
| yes | 476 (4.5) | 446 (4.5) | 30 (6.0) | 0.102 | 1.373 (0.938-2.069) |
| no | 9996 (95.5) | 9529 (95.5) | 467 (94.0) |  |  |
| CS |  |  |  |  |  |
| yes | 6353 (60.7) | 5867 (58.8) | 486 (97.8) | <0.001 | 30.936 (16.996-56.308) |
| no | 4119 (39.3) | 4108 (41.2) | 11 (2.2) |  |  |
| Intrauterine distress | |  |  |  |  |
| yes | 757 (7.2) | 748 (7.5) | 9 (1.8) | <0.001 | 0.228 (0.117-0.442) |
| no | 9715 (92.8) | 9227 (92.5) | 488 (98.2) |  |  |
| FGR |  |  |  |  |  |
| yes | 66 (0.6) | 58 (0.6) | 8 (1.6) | 0.005 | 2.797 (1.328-5.891) |
| no | 10406 (99.4) | 9917 (99.4) | 489 (98.4) |  |  |
| SGA |  |  |  |  |  |
| yes | 53 (0.5) | 46 (0.5) | 7 (1.4) | 0.004 | 3.084 (1.385-6.865) |
| no | 10419 (99.5) | 9929 (99.5) | 490 (98.6) |  |  |
| Anemia |  |  |  |  |  |
| yes | 935 (8.9) | 837 (8.4) | 98 (19.7) | <0.001 | 2.682 (2.126-3.382) |
| no | 9537 (91.1) | 9138 (91.6) | 399 (80.3) |  |  |
| Polyhydramnios | |  |  |  |  |
| yes | 144 (1.4) | 130 (1.3) | 14 (2.8) | 0.005 | 2.195 (1.255-3.838) |
| no | 10328 (98.6) | 9845 (98.7) | 483 (97.2) |  |  |
| Oligohydramnios | |  |  |  |  |
| yes | 373 (3.6) | 369 (3.7) | 4 (0.8) | 0.001 | 0.211 (0.079-0.568) |
| no | 10099 (96.4) | 9606 (96.3) | 493 (99.2) |  |  |
| Amniotic fluid contamination | |  |  |  |  |
| yes | 66 (0.6) | 66 (0.7) | 0 (0.0) | 0.069 | UA |
| no | 10406 (99.4) | 9909 (99.3) | 497 (100) |  |  |
| **Abbreviations:** PROM: premature rupture of membranes, PTB: preterm births, LBW: low birth weight, PPH: postpartum hemorrhage, CS: cesarean section, FGR: fetal growth restriction, SGA: Small for gestational age. UA: Due to the case being 0, the value was unavailable. | | | | | |

**Table S8. Comparison of perinatal complications between singleton and twin pregnancies in GDM group.**

|  | **GDM group (n=20791, %)** | **Subgroups** | | |  |
| --- | --- | --- | --- | --- | --- |
| **Variables** |  | **Singleton pregnancies (n=20349, %)** | **Twin pregnancies (n=442, %)** | ***P* value** | **OR (95%CI)**  **(twin vs singleton)** |
| Placenta previa | |  |  |  |  |
| yes | 512 (2.5) | 497 (2.4) | 15 (3.4) | 0.202 | 1.403 (0.832-2.366) |
| no | 20279 (97.7) | 19852 (97.6) | 427 (96.6) |  |  |
| PROM |  |  |  |  |  |
| yes | 3286 (15.8) | 3233 (15.9) | 53 (12.0) | 0.026 | 0.721 (0.540-0.963) |
| no | 17505 (84.2) | 17116 (84.1) | 389 (88.0) |  |  |
| Placental abruption | |  |  |  |  |
| yes | 180 (0.9) | 172 (0.8) | 8 (1.8) | 0.030 | 2.162 (1.058-4.421) |
| no | 20611 (99.1) | 20177 (99.2) | 434 (98.2) |  |  |
| Placental implantation | |  |  |  |  |
| yes | 204 (1.0) | 197 (1.0) | 7 (1.6) | 0.194 | 1.646 (0.770-3.519) |
| no | 20587 (99.0) | 20152 (99.0) | 435 (98.4) |  |  |
| Cord protrusion | |  |  |  |  |
| yes | 43 (0.2) | 41 (0.2) | 2 (0.5) | 0.250 | 2.251 (0.543-9.337) |
| no | 20748 (99.8) | 20308 (99.8) | 440 (99.5) |  |  |
| Cord entanglement | |  |  |  |  |
| yes | 2846 (13.7) | 2821 (13.9) | 25 (5.7) | <0.001 | 0.373 (0.248-0.559) |
| no | 17945 (86.3) | 17528 (86.1) | 417 (94.3) |  |  |
| Cord torsion | |  |  |  |  |
| yes | 1718 (8.3) | 1697 (8.3) | 21 (4.8) | 0.007 | 0.548 (0.353-0.852) |
| no | 19073 (91.7) | 18652 (91.7) | 421 (95.2) |  |  |
| Rupture of uterus | |  |  |  |  |
| yes | 295 (1.4) | 287 (1.4) | 8 (1.8) | 0.482 | 1.289 (0.634-2.618) |
| no | 20496 (98.6) | 20062 (98.6) | 434 (98.2) |  |  |
| Postpartum embolism | |  |  |  |  |
| yes | 15 (0.1) | 14 (0.1) | 1 (0.2) | 0.223 | 3.294 (0.432-25.102) |
| no | 20776 (99.9) | 20335 (99.9) | 441 (99.8) |  |  |
| Postpartum thrombosis | |  |  |  |  |
| yes | 91 (0.4) | 86 (0.4) | 5 (1.1) | 0.026 | 2.696 (1.089-6.675) |
| no | 20700 (99.6) | 20263 (99.6) | 437 (98.9) |  |  |
| PTB |  |  |  |  |  |
| yes | 1879 (9.0) | 1574 (7.7) | 305 (69.0) | <0.001 | 26.556 (21.568-32.697) |
| no | 18912 (91.0) | 18775 (92.3) | 137 (31.0) |  |  |
| Macrosomia | |  |  |  |  |
| yes | 871 (4.3) | 871 (4.2) | 0 (0) | <0.001 | UA |
| no | 19920 (95.8) | 19478 (95.7) | 442 (100.0) |  |  |
| LBW |  |  |  |  |  |
| yes | 64 (0.3) | 61 (0.3) | 3 (0.7) | 0.155 | 2.273 (0.710-7.271) |
| no | 20727 (99.7) | 20288 (99.7) | 439 (99.3) |  |  |
| Neonatal asphyxia | |  |  |  |  |
| yes | 112 (0.5) | 101 (0.5) | 11 (2.5) | <0.001 | 5.117 (2.726-9.603) |
| no | 20679 (99.5) | 20248 (99.5) | 431 (97.1) |  |  |
| PPH |  |  |  |  |  |
| yes | 786 (3.8) | 765 (3.8) | 21 (4.8) | 0.279 | 1.277 (0.819-1.991) |
| no | 20005 (96.2) | 19584 (96.2) | 421 (95.2) |  |  |
| CS |  |  |  |  |  |
| yes | 10979 (52.8) | 10548 (51.8) | 431 (97.5) | <0.001 | 36.407 (19.999-66.277) |
| no | 9812 (47.2) | 9801 (48.2) | 11 (2.5) |  |  |
| Intrauterine distress | |  |  |  |  |
| yes | 1276 (6.1) | 1264 (6.2) | 12 (2.7) | 0.002 | 0.421 (0.237-0.750) |
| no | 19515 (93.9) | 19085 (93.8) | 430 (97.3) |  |  |
| FGR |  |  |  |  |  |
| yes | 28 (0.1) | 25 (0.1) | 3 (.7) | 0.021 | 5.556 (1.671-18.469) |
| no | 20783 (99.9) | 20324 (99.9) | 439 (99.3) |  |  |
| SGA |  |  |  |  |  |
| yes | 47 (0.2) | 43 (0.2) | 4 (0.9) | 0.017 | 4.313 (1.541-12.067) |
| no | 20744 (99.8) | 20306 (99.8) | 438 (99.1) |  |  |
| Anemia |  |  |  |  |  |
| yes | 1635 (7.9) | 1570 (7.7) | 65 (14.7) | <0.001 | 2.062 (1.577-2.697) |
| no | 19156 (92.1) | 18779 (92.3) | 377 (85.3) |  |  |
| Polyhydramnios | |  |  |  |  |
| yes | 461 (2.2) | 443 (2.2) | 18 (4.1) | 0.007 | 1.908 (1.179-3.086) |
| no | 20330 (97.8) | 19906 (97.8) | 424 (95.9) |  |  |
| Oligohydramnios | |  |  |  |  |
| yes | 548 (2.6) | 540 (2.7) | 8 (1.5) | 0.273 | 0.676 (0.334-1.368) |
| no | 20243 (97.4) | 19809 (97.3) | 434 (98.2) |  |  |
| Amniotic fluid contamination | |  |  |  |  |
| yes | 139 (0.7) | 139 (0.7) | 0 (0) | 0.081 | UA |
| no | 20552 (99.3) | 20210 (99.3) | 442 (100) |  |  |
| **Abbreviations:** PROM: premature rupture of membranes, PTB: preterm births, LBW: low birth weight, PPH: postpartum hemorrhage, CS: cesarean section, FGR: fetal growth restriction, SGA: Small for gestational age. UA: Due to the case being 0, the value was unavailable. | | | | | |

**Table S9.** **Comparison of perinatal complications between singleton and twin pregnancies in HDP-GDM group**.

|  | **HDP-GDM group (n=3228, %)** | **Subgroups** | | | | | |  | |  |
| --- | --- | --- | --- | --- | --- | --- | --- | --- | --- | --- |
| **Variables** |  | **Singleton pregnancies (n=3081, %)** | | **Twin pregnancies (n=147, %)** | | ***P* value** | | **OR (95%CI)**  **(twin vs singleton)** | |  |
| Placenta previa | | |  | |  | |  | |  | |
| yes | 59 (1.8) | 53 (1.7) | | 6 (4.1) | | 0.037 | | 2.431 (1.028-5.751) | |  |
| no | 3169 (98.2) | 3028 (98.3) | | 141 (95.9) | |  | |  | |  |
| PROM |  |  | |  | |  | |  | |  |
| yes | 458 (14.2) | 444 (14.4) | | 14 (9.5) | | 0.097 | | 0.625 (0.357-1.094) | |  |
| no | 2770 (85.8) | 2637 (85.6) | | 133 (90.5) | |  | |  | |  |
| Placental abruption | | |  | |  | |  | |  | |
| yes | 49 (1.5) | 44 (1.4) | | 5 (3.4) | | 0.056 | | 2.430 (0.949-6.223) | |  |
| no | 3179 (98.5) | 3037 (98.6) | | 142 (96.6) | |  | |  | |  |
| Placental implantation | | |  | |  | |  | |  | |
| yes | 34 (1.1) | 32 (1.0) | | 2 (1.4) | | 0.709 | | 1.314 (0.312-5.537) | |  |
| no | 3194 (98.9) | 3049 (99.0) | | 145 (98.6) | |  | |  | |  |
| Cord protrusion | | |  | |  | |  | |  | |
| yes | 6 (0.2) | 6 (0.2) | | 0 (0) | | 0.592 | | UA | |  |
| no | 3222 (99.8) | 3075 (99.8) | | 147 (100.0) | |  | |  | |  |
| Cord entanglement | | |  | |  | |  | |  | |
| yes | 383 (11.9) | 373 (12.1) | | 10 (6.8) | | 0.052 | | 0.530 (0.276-1.016) | |  |
| no | 2845 (88.1) | 2708 (87.9) | | 137 (93.2) | |  | |  | |  |
| Cord torsion | | |  | |  | |  | |  | |
| yes | 240 (7.4) | 237 (7.7) | | 3 (2.0) | | 0.011 | | 0.250 (0.079-0.790) | |  |
| no | 2988 (92.6) | 2844 (92.3) | | 144 (98.0) | |  | |  | |  |
| Rupture of uterus | | |  | |  | |  | |  | |
| yes | 34 (1.1) | 31 (1.0) | | 3 (2.0) | | 0.230 | | 2.050 (0.619-6.784) | |  |
| no | 3194 (98.9) | 3050 (99.0) | | 144 (98.0) | |  | |  | |  |
| Postpartum embolism | | |  | |  | |  | |  | |
| yes | 6 (0.2) | 4 (0.1) | | 2 (1.4) | | 0.001 | | 10.610 (1.928-58.403) | |  |
| no | 3222 (99.8) | 3077 (99.9) | | 145 (98.6) | |  | |  | |  |
| Postpartum thrombosis | | |  | |  | |  | |  | |
| yes | 15 (0.5) | 13 (0.4) | | 2 (1.4) | | 0.102 | | 3.255 (0.728-14.559) | |  |
| no | 3213 (99.5) | 3068 (99.6) | | 145 (98.6) | |  | |  | |  |
| PTB |  |  | |  | |  | |  | |  |
| yes | 565 (17.5) | 461 (15.0) | | 104 (70.7) | | <0.001 | | 13.746 (9.505-19.878) | |  |
| no | 2663 (82.5) | 2620 (85.0) | | 43 (29.3) | |  | |  | |  |
| Macrosomia |  |  | |  | |  | |  | |  |
| yes | 131 (4.1) | 131 (4.3) | | 0 (0) | | 0.011 | | UA | |  |
| no | 3097 (95.9) | 2950 (95.7) | | 147 (100.0) | |  | |  | |  |
| LBW |  |  | |  | |  | |  | |  |
| yes | 28 (0.9) | 27 (0.9) | | 1 (0.7) | | 0.802 | | 0.775 (0.105-5.741) | |  |
| no | 3200 (99.1) | 3054 (99.1) | | 146 (99.3) | |  | |  | |  |
| Neonatal asphyxia | | |  | |  | |  | |  | |
| yes | 37 (1.1) | 35 (1.1) | | 2 (1.4) | | 0.803 | | 1.200 (0.286-5.039) | |  |
| no | 3191 (98.9) | 3046 (98.9) | | 145 (98.6) | |  | |  | |  |
| PPH |  |  | |  | |  | |  | |  |
| yes | 127 (3.9) | 120 (3.9) | | 7 (4.8) | | 0.597 | | 1.234 (0.565-2.693) | |  |
| no | 3101 (96.1) | 2961 (96.1) | | 140 (95.2) | |  | |  | |  |
| CS |  |  | |  | |  | |  | |  |
| yes | 2189 (67.8) | 2045 (66.4) | | 144 (98.0) | | <0.001 | | 24.317 (7.732-76.472) | |  |
| no | 1039 (32.2) | 1036 (33.6) | | 3 (2.0) | |  | |  | |  |
| Intrauterine distress | | |  | |  | |  | |  | |
| yes | 202 (6.3) | 198 (6.4) | | 4 (2.7) | | 0.070 | | 0.407 (0.149-1.112) | |  |
| no | 3026 (93.7) | 2883 (93.6) | | 143 (97.3) | |  | |  | |  |
| FGR |  |  | |  | |  | |  | |  |
| yes | 22 (0.7) | 20 (0.6) | | 2 (1.4) | | 0.306 | | 2.111 (0.489-9.118) | |  |
| no | 3206 (99.3) | 3061 (99.4) | | 145 (98.6) | |  | |  | |  |
| SGA |  |  | |  | |  | |  | |  |
| yes | 28 (0.9) | 27 (0.9) | | 1 (0.7) | | 0.802 | | 0.775 (0.105-5.741) | |  |
| no | 3200 (99.1) | 3054 (99.1) | | 146 (99.3) | |  | |  | |  |
| Anemia |  |  | |  | |  | |  | |  |
| yes | 228 (7.1) | 206 (6.7) | | 22 (15.0) | | <0.001 | | 2.456 (1.528-3.949) | |  |
| no | 3000 (92.9) | 2875 (93.3) | | 125 (85.0) | |  | |  | |  |
| Polyhydramnios | | |  | |  | |  | |  | |
| yes | 59 (1.8) | 56 (1.8) | | 3 (2.0) | | 0.844 | | 1.125 (0.348-3.639) | |  |
| no | 3169 (98.2) | 3025 (98.2) | | 144 (98.0) | |  | |  | |  |
| Oligohydramnios | | |  | |  | |  | |  | |
| yes | 94 (2.9) | 93 (3.0) | | 1 (0.7) | | 0.100 | | 0.220 (0.030-1.590) | |  |
| no | 3134 (97.1) | 2988 (97.0) | | 146 (99.3) | |  | |  | |  |
| Amniotic fluid contamination | | |  | |  | |  | |  | |
| yes | 21 (0.7) | 19 (0.6) | | 2 (1.4) | | 0.273 | | 2.223 (0.513-9.634) | |  |
| no | 3207 (99.3) | 3062 (99.4) | | 145 (98.6) | |  | |  | |  |
| **Abbreviations:** PROM: premature rupture of membranes, PTB: preterm births, LBW: low birth weight, PPH: postpartum hemorrhage, CS: cesarean section, FGR: fetal growth restriction, SGA: Small for gestational age. UA: Due to the case being 0, the value was unavailable. | | | | | | | | | | |

**Table S10. Comparison of perinatal complications between different HDP/GDM groups in singleton pregnancies.**

| **Variables** | **Singleton pregnancies**  **(n=128593, %)** | **Groups** | | | | | | | | | |  |
| --- | --- | --- | --- | --- | --- | --- | --- | --- | --- | --- | --- | --- |
|  |  | **Non-HDP-GDM group (n=95188, %)** | | **HDP group (n=9975, %)** | | **GDM group (n=20349, %)** | | **HDP-GDM group (n=3081, %)** | | ***P* value** | |  |
| Placenta previa | | |  | |  | |  | |  | |  | |
| yes | 2887 (2.2) | 2167 (2.3) | | 170 (1.7) | | 497 (2.4) | | 53 (1.7) | | <0.001 | |  |
| no | 125706 (97.8) | 93021 (97.7) | | 9805 (98.3) | | 19853 (97.6) | | 3028 (98.3) | |  | |  |
| PROM | | |  | |  | |  | |  | |  | |
| yes | 23427 (18.2) | 18125 (19.0) | | 1625 (16.3) | | 3233 (13.8) | | 444 (14.4) | | <0.001 | |  |
| no | 105166 (81.8) | 77083 (81.0) | | 8350 (83.7) | | 17116 (84.1) | | 2637 (85.6) | |  | |  |
| Placental abruption | | |  | |  | |  | |  | |  | |
| yes | 1010 (0.8) | 674 (0.7) | | 120 (1.2) | | 172 (0.8) | | 44 (1.4) | | <0.001 | |  |
| no | 127583 (99.2) | 94514 (99.3) | | 9855 (98.8) | | 20177 (99.2) | | 3037 (98.6) | |  | |  |
| Placental implantation | | |  | |  | |  | |  | |  | |
| yes | 919 (0.7) | 601 (0.6) | | 89 (0.9) | | 197 (1.0) | | 32 (1.0) | | <0.001 | |  |
| no | 127674 (99.3) | 94587 (99.4) | | 9886 (99.1) | | 20152 (99.0) | | 3049 (99.0) | |  | |  |
| Cord protrusion | | |  | |  | |  | |  | |  | |
| yes | 186 (0.1) | 1234 (0.1) | | 16 (0.2) | | 41 (0.2) | | 6 (0.2) | | 0.078 | |  |
| no | 128407 (99.9) | 95065 (99.9) | | 9959 (99.8) | | 20308 (99.8) | | 3075 (99.8) | |  | |  |
| Cord entanglement | | |  | |  | |  | |  | |  | |
| yes | 14825 (11.5) | 10609 (11.1) | | 1022 (10.2) | | 2821 (13.9) | | 373 (12.1) | | <0.001 | |  |
| no | 113768 (88.5) | 84579 (88.9) | | 8953 (89.8) | | 17528 (86.1) | | 2708 (87.9) | |  | |  |
| Cord torsion | | |  | |  | |  | |  | |  | |
| yes | 9579 (7.4) | 6932 (7.3) | | 713 (7.1) | | 1697 (8.3) | | 237 (7.7) | | <0.001 | |  |
| no | 119014 (92.6) | 88256 (92.7) | | 9262 (92.9) | | 18652 (91.7) | | 2844 (92.3) | |  | |  |
| Rupture of uterus | | |  | |  | |  | |  | |  | |
| yes | 1523 (1.2) | 1148 (1.2) | | 57 (0.62) | | 287 (1.4) | | 3 (1.0) | | <0.001 | |  |
| no | 127070 (98.8) | 94040 (98.8) | | 9918 (99.4) | | 20062 (98.6) | | 3050 (99.0) | |  | |  |
| Postpartum embolism | | |  | |  | |  | |  | |  | |
| yes | 61 (0) | 37 (0) | | 6 (0.1) | | 14 (0.1) | | 4 (0.1) | | 0.044 | |  |
| no | 128532 (100) | 95151 (100) | | 9969 (100) | | 20335 (99.9) | | 3077 (99.9) | |  | |  |
| Postpartum thrombosis | | |  | |  | |  | |  | |  | |
| yes | 347 (0.3) | 223 (0.2) | | 25(0.3) | | 86 (0.4) | | 13 (0.4) | | <0.001 | |  |
| no | 128246 (99.7) | 94965 (99.8) | | 9950 (99.7) | | 20263 (99.6) | | 2068 (99.6) | |  | |  |
| PTB |  |  | |  | |  | |  | |  | |  |
| yes | 8957 (7.0) | 5695 (6.0) | | 1227 (12.3) | | 1574 (7.7) | | 461 (15.0) | | <0.001 | |  |
| no | 119636 (93.0) | 89493 (94.0) | | 8748 (87.7) | | 18775 (92.3) | | 2620 (85.0) | |  | |  |
| Macrosomia |  |  | |  | |  | |  | |  | |  |
| yes | 5444 (4.2) | 4049 (4.3) | | 393 (3.9) | | 871 (4.3) | | 131 (4.3) | | <0.001 | |  |
| no | 123149 (95.8) | 91139 (95.7) | | 9582 (96.1) | | 19478 (95.7) | | 2950 (95.7) | |  | |  |
| LBW |  |  | |  | |  | |  | |  | |  |
| yes | 357 (0.3) | 198 (0.2) | | 71 (0.7) | | 61 (0.3) | | 27 (0.9) | | <0.001 | |  |
| no | 128236 (99.7) | 94990 (99.8) | | 9904 (99.3) | | 20288 (99.7) | | 3054 (99.1) | |  | |  |
| Neonatal asphyxia | | |  | |  | |  | |  | |  | |
| yes | 681 (0.5) | 461 (0.5) | | 84 (0.8) | | 101 (0.5) | | 35 (1.1) | | <0.001 | |  |
| no | 127912 (99.5) | 94727 (99.5) | | 9891 (99.5) | | 20248 (99.5) | | 3046 (98.9) | |  | |  |
| PPH |  |  | |  | |  | |  | |  | |  |
| yes | 4790 (3.7) | 3459 (3.6) | | 446 (4.5) | | 765 (3.8) | | 120 (3.9) | | <0.001 | |  |
| no | 93370 (96.4) | 91729 (96.4) | | 9529 (95.5) | | 19584 (96.2) | | 2961 (96.1) | |  | |  |
| CS |  |  | |  | |  | |  | |  | |  |
| yes | 61213 (47.6) | 42753 (44.9) | | 5867 (58.8) | | 10548 (51.8) | | 2045 (66.4) | | <0.001 | |  |
| no | 67380 (52.4) | 52435 (55.1) | | 41108 (41.2) | | 9801 (48.2) | | 1036 (33.6) | |  | |  |
| Intrauterine distress | | |  | |  | |  | |  | |  | |
| yes | 8766 (6.8) | 6556 (6.9) | | 748 (7.5) | | 1264 (6.2) | | 198 (6.4) | | <0.001 | |  |
| no | 119827 (93.2) | 88632 (93.1) | | 9227 (92.5) | | 19085 (93.8) | | 2883 (93.6) | |  | |  |
| FGR |  |  | |  | |  | |  | |  | |  |
| yes | 168 (0.1) | 65 (0.1) | | 58 (0.6) | | 25 (0.1) | | 20 (0.6) | | <0.001 | |  |
| no | 128425 (99.9) | 95123 (99.9) | | 9917 (99.4) | | 20324 (99.9) | | 3061 (99.4) | |  | |  |
| SGA |  |  | |  | |  | |  | |  | |  |
| yes | 225 (0.2) | 109 (0.1) | | 46 (0.5) | | 43 (0.2) | | 27 (0.9) | | <0.001 | |  |
| no | 128368 (99.8) | 95079 (99.9) | | 9929 (99.5) | | 20306 (99.8) | | 3054 (99.1) | |  | |  |
| Anemia |  |  | |  | |  | |  | |  | |  |
| yes | 11864 (9.2) | 9251 (9.7) | | 837 (8.4) | | 1570 (7.7) | | 206 (6.7) | | <0.001 | |  |
| no | 116729 (90.8) | 85937 90.3) | | 9138 (91.6) | | 18779 (92.3) | | 2875 (93.3) | |  | |  |
| Polyhydramnios |  |  | |  | |  | |  | |  | |  |
| yes | 1911 (1.5) | 1282 (1.3) | | 130 (1.3) | | 443 (2.2) | | 56 (1.8) | | <0.001 | |  |
| no | 126682 (98.5) | 93906 (98.7) | | 9845 (98.7) | | 19906 (97.8) | | 3025 (98.2) | |  | |  |
| Oligohydramnios |  |  | |  | |  | |  | |  | |  |
| yes | 4421 (3.4) | 3419 (3.6) | | 369 (3.7) | | 540 (2.7) | | 93 (3.0) | | <0.001 | |  |
| no | 124172 (96.6) | 91769 (96.4) | | 9606 (96.3) | | 19809 (97.3) | | 2988 (97.0) | |  | |  |
| Amniotic fluid contamination | | |  | |  | |  | |  | |  | |
| yes | 882 (0.7) | 658 (0.7) | | 66 (0.7) | | 139 (0.7) | | 19 (0.6) | | 0.951 | |  |
| no | 127711 (99.3) | 94530 (99.3) | | 9909 (99.3) | | 20210 (99.3) | | 3062 (99.4) | |  | |  |
| **Abbreviations:** PROM: premature rupture of membranes, PTB: preterm births, LBW: low birth weight, PPH: postpartum hemorrhage, CS: cesarean section, FGR: fetal growth restriction, SGA: Small for gestational age. | | | | | | | | | | | | |

**Table S11. Comparison of perinatal complications between different HDP/GDM groups in twin pregnancies.**

| **Variables** | **Twin pregnancies**  **(n=2803, %)** | **Groups** | | | | |
| --- | --- | --- | --- | --- | --- | --- |
|  |  | **Non-HDP-GDM group（n=1717, %）** | **HDP group (n=497, %)** | **GDM group (n=442, %)** | **HDP-GDM group (n=147, %)** | ***P* value** |
| Placenta previa | |  |  |  |  |  |
| yes | 96 (3.4) | 56 (3.3) | 19 (3.8) | 15 (3.4) | 6 (4.1) | 0.903 |
| no | 2707 (96.6) | 1661 (96.7) | 478 (96.2) | 427 (96.6) | 141 (95.9) |  |
| PROM |  |  |  |  |  |  |
| yes | 351 (12.5) | 231 (13.5) | 53 (10.7) | 53 (12.0) | 14 (9.5) | 0.236 |
| no | 2452 (87.5) | 1486 (86.5) | 444 (89.3) | 389 (88.0) | 133 (90.5) |  |
| Placental abruption | |  |  |  |  |  |
| yes | 49 (1.7) | 29 (1.7) | 7 (1.4) | 8 (1.8) | 5 (3.4) | 0.437 |
| no | 2754 (98.3) | 1688 (98.3) | 490 (98.6) | 434 (98.2) | 142 (96.6) |  |
| Placental implantation | |  |  |  |  |  |
| yes | 47 (1.7) | 26 (1.5) | 12 (2.4) | 7 (1.6) | 2 (1.4) | 0.567 |
| no | 2756 (98.3) | 1691 (98.5) | 485 (97.6) | 435 (98.4) | 145 (98.6) |  |
| Cord protrusion |  |  |  |  |  |  |
| yes | 6 (0.2) | 4 (0.2) | 0 (0) | 2 (0.5) | 0 (0) | 0.460 |
| no | 2797 (99.8) | 1713 (99.8) | 497 (100) | 440 (99.5) | 147 (100) |  |
| Cord entanglement | |  |  |  |  |  |
| yes | 152 (5.4) | 85 (5.0) | 32 (6.4) | 25 (5.7) | 10 (6.8) | 0.505 |
| no | 2651 (94.6) | 1632 (95.0) | 465 (93.6) | 417 (94.3) | 137 (93.2) |  |
| Cord torsion |  |  |  |  |  |  |
| yes | 112 (4.0) | 67(3.9) | 21 (4.2) | 21 (4.8) | 3 (2.0) | 0.526 |
| no | 26914 (96.0) | 1650 (96.1) | 476 (95.8) | 421 (95.2) | 144 (98.0) |  |
| Rupture of uterus | |  |  |  |  |  |
| yes | 32 (1.1) | 16 (0.9) | 5 (1.0) | 8 (1.8) | 3 (2.0) | 0.314 |
| no | 2771 (98.9) | 1701 (99.1) | 492 (99.0) | 434 (98.2) | 144 (98.0) |  |
| Postpartum embolism | |  |  |  |  |  |
| yes | 8 (0.3) | 4 (0.2) | 1 (0.2) | 1 (0.2) | 2 (1.4) | 0.097 |
| no | 2795 (99.7) | 1713 (99.8) | 496 (99.8) | 441 (99.8) | 145 (98.6) |  |
| Postpartum thrombosis | |  |  |  |  |  |
| yes | 18 (0.6) | 10 (0.6) | 1 (0.6) | 5 (1.1) | 2 (1.4) | 0.216 |
| no | 145 (98.6) | 1707 (99.4) | 496 (99.8) | 437 (98.9) | 145 (98.6) |  |
| PTB |  |  |  |  |  |  |
| yes | 1842 (65.7) | 1088 (63.4) | 345 (69.4) | 305 (69.0) | 104 (70.7) | 0.012 |
| no | 961 (34.3) | 629 (36.6) | 152 (30.6) | 137 (31.0) | 43 (29.3) |  |
| Macrosomia |  |  |  |  |  |  |
| yes | 2 (0.1) | 2 (0.1) | 0 (0) | 0 (0) | 0 (0) | 0.737 |
| no | 2801 (99.9) | 1715 (99.9) | 497 (100) | 442 (100) | 147 (5.2) |  |
| LBW |  |  |  |  |  |  |
| yes | 27 (1.0) | 19 (1.1) | 4 (0.8) | 3 (0.7) | 1 (0.7) | 0.802 |
| no | 2776 (99.0) | 1698 (98.9) | 493 (99.2) | 439 (99.3) | 146 (99.3) |  |
| Neonatal asphyxia | |  |  |  |  |  |
| yes | 74 (2.6) | 51 (3.0) | 10 (2.0) | 11 (2.5) | 2 (1.4) | 0.481 |
| no | 2729 (97.4) | 1666 (97.0) | 487 (98.0) | 431 (97.5) | 145 (98.6) |  |
| PPH |  |  |  |  |  |  |
| yes | 134 (4.8) | 76 (4.4) | 30 (6.0) | 21 (4.8) | 7 (4.8) | 0.533 |
| no | 2669 (95.2) | 1641 (95.6) | 467 (94.0) | 421 (95.2) | 140 (95.2) |  |
| CS |  |  |  |  |  |  |
| yes | 2704 (96.5) | 1643 (95.7) | 486 (97.8) | 431 (97.5) | 144 (98.0) | 0.047 |
| no | 99 (3.5) | 74 (4.3) | 11 (2.2) | 11 (2.5) | 3 (2.0) |  |
| Intrauterine distress | |  |  |  |  |  |
| yes | 45 (1.6) | 20 (1.2) | 9 (1.8) | 12 (2.7) | 4 (2.7) | 0.077 |
| no | 2758 (98.4) | 1697 (98.8) | 488 (98.2) | 430 (97.3) | 143 (97.3) |  |
| FGR |  |  |  |  |  |  |
| yes | 69 (2.5) | 35 (2.0) | 19 (3.8) | 10 (2.3) | 5 (3.4) | 0.125 |
| no | 2734 (97.5) | 1682 (98.0) | 478 (96.2) | 432 (97.7) | 142 (96.6) |  |
| SGA |  |  |  |  |  |  |
| yes | 24 (0.9) | 12 (0.7) | 7 (1.4) | 4 (0.9) | 1 (0.7) | 0.503 |
| no | 2779 (99.1) | 1705 (99.3) | 490 (98.6) | 438 (99.1) | 146 (99.3) |  |
| Anemia |  |  |  |  |  |  |
| yes | 481 (17.2) | 296 (17.2) | 98 (19.7) | 65 (14.7) | 22 (15.0) | 0.198 |
| no | 2322 (82.8) | 1421 82.8) | 399 (80.3) | 377 (85.3) | 125 (85.0) |  |
| Polyhydramnios | |  |  |  |  |  |
| yes | 76 (2.7) | 41 (2.4) | 14 (2.8) | 18 (4.1) | 3 (2.0) | 0.255 |
| no | 2727 (97.3) | 1676 (97.6) | 483 (97.2) | 424 (95.9) | 144 (98.0) |  |
| Oligohydramnios | |  |  |  |  |  |
| yes | 31 (1.1) | 18 (1.0) | 4 (0.8) | 8 (1.8) | 1 (0.7) | 0.438 |
| no | 2772 (98.9) | 1699 (99.0) | 493 (99.2) | 434 (98.2) | 146 (99.3) |  |
| Amniotic fluid contamination | |  |  |  |  |  |
| yes | 6 (0.2) | 4 (0.2) | 0 (0) | 0 (0) | 2 (1.4) | 0.028 |
| no | 2797 (99.8) | 1713 (99.8) | 497 (100) | 442 (100) | 145 (98.6) |  |
| **Abbreviations:** PROM: premature rupture of membranes, PTB: preterm births, LBW: low birth weight, PPH: postpartum hemorrhage, CS: cesarean section, FGR: fetal growth restriction, SGA: Small for gestational age. | | | | | | |
